# Heritability of the *Pinus radiata* root microbiome

**DOI:** 10.3389/fpls.2026.1793374

**Published:** 2026-04-02

**Authors:** Natalie J Graham, Gancho Slavov, Steve A. Wakelin, Jaroslav Klápště, Nicola J. Day

**Affiliations:** 1 Bioeconomy Science Institute, Rotorua, New Zealand; 2 School of Biological Sciences, Victoria University of Wellington, Wellington, New Zealand; 3 Pacific Northwest Tree Improvement Research Cooperative, Department of Forest Engineering, Resources, and Management, College of Forestry, Oregon State University, Corvallis, OR, United States; 4 Tree Breeding Australia Ltd., Mount Gambier, Australia

**Keywords:** bacteria, core microbiome, fungi, heritability, host genetics, plant-microbe interactions, rhizosphere

## Abstract

**Introduction:**

Both evolutionary history and recent breeding selections can influence plant microbiomes, with closely-related individuals often having more similar microbiomes. *Pinus radiata* D.Don is an emerging conifer model species for investigating tree-microbiome interactions. However, little is known about how the *P. radiata* microbiome covaries with host genotype, especially beyond the seedling stage.

**Methods:**

We sampled the root microbiome of 528 individual *P. radiata* trees (age = 9 yrs), comprising four clonal copies each of 132 host genotypes from 28 full-sib families, from a clonal breeding trial in New Zealand. We determined whether variations in the bacterial and fungal root microbiomes were associated with host ancestry (i.e., provenance), family, and genotype.

**Results:**

Host family was associated with fungal but not bacterial root microbiome composition, whereas broader ancestry and individual genotype within families had no detectable effect on either bacterial or fungal microbiome composition. While core (in ≥80% samples) amplicon sequence variants (ASVs) typically had negligible heritability, the relative abundances of 83 bacterial and 13 fungal non-core ASVs had low to moderate broad-sense heritabilities (0.2 to 0.46). Host genetic effects associated with abundances of heritable ASVs were primarily non-additive and likely involve complex gene interactions.

**Discussion:**

Our study revealed subtle host family effects for the root fungal microbiome of *P. radiata*, with several heritable bacterial and fungal ASVs. This study broadens our understanding of host genetic influences on the composition of the root microbiome of *P. radiata* and indicates there are both opportunities and challenges for including microbiome-related traits in tree breeding programmes.

## Introduction

Microbiome assembly is influenced by both microbial and plant traits which shape plant-microbe interactions. Both the evolutionary history and recent breeding selections of the host can influence the community composition of the plant microbiome, with closely-related host species or individuals with similar traits often having more similar microbiomes (Lim and Bordenstein, 2020). Host traits linked with microbiome associations can include the secretion profile of secondary metabolites (Bais et al., 2006; Mönchgesang et al., 2016), differences in disease resistance (Mauchline et al., 2015; Mendes et al., 2018), and other heritable physiological mechanisms under host genetic control (Szoboszlay et al., 2015). Variations in plant phenotypes have been linked to variations in the composition of the microbiome (Henry et al., 2021; Lasa et al., 2024), and indirect microbiome manipulation via selection of host traits is being explored for sustainable and enduring mitigation of biotic and abiotic stresses. For example, breeding for microbiome-related or “M genes” has been suggested to improve disease resistance in rice (Cernava, 2024; Su et al., 2024; Zhan and Wang, 2024). Unfortunately, many studies that demonstrate positive microbial effects on plant hosts are not performed under real-world conditions, limiting their application beyond experimental settings (Sessitsch et al., 2019). However, the complexity and variability of field conditions compared with tightly controlled lab environments poses challenges for assessing plant-microbe interactions.

Quantitative traits are often subject to strong environmental influences and are thus usually of low to moderate heritability (Klápště et al., 2020). The heritability for quantitative microbiome-related traits, such as microbial diversity and relative abundances of taxa, also tends to be low (Bruijning et al., 2023), even for core microbiome species that are present in most host individuals. Knowledge of microbiome heritability in plant systems has largely been restricted to roots and leaves in a few agricultural species, including sorghum, maize, and switchgrass (e.g., Deng et al., 2021; He et al., 2024; Sutherland et al., 2022; Wallace et al., 2018; Walters et al., 2018). In these studies, heritability estimates for various microbiome metrics were predominantly low to moderate with large standard errors, reflecting the modest experimental sample sizes and relatively weak genetic control. Studies in non-agricultural species are limited, with examples including *Populus* spp (Lamit et al., 2016; Schweitzer et al., 2008), *Boechera stricta* (Wagner et al., 2016) and *Ipomoea hederacea* (Boyle et al., 2024). Further studies in well-structured host populations are needed to address the knowledge gap of how host-microbial interactions vary with different host genetics in non-crop species, particularly in long-lived forest trees.

The widely-distributed conifer *Pinus radiata* D.Don is emerging as a model species for investigating tree-microbiome interactions on needles (S.L. Addison et al., 2023), pollen (Armstrong et al., 2024), and roots (Sarah L. Addison et al., 2023; Gallart et al., 2018; Rúa and Hoeksema, 2024). With existing genetic and genomic resources (Graham et al., 2022; Sturrock et al., 2025), extensive breeding trial networks, and comprehensive pedigrees (McLean et al., 2023; Paget, 2022), this species is well-positioned for advancing our understanding of tree-microbiome interactions in conifers. It is one of the few non-agricultural species in which host genotype effects on the overall composition of the root microbiome have been demonstrated, albeit in only two genotypes known to vary in their response to nitrogen (Gallart et al., 2018). Another study with a sufficiently large population (n = 472) for quantitative genetics analysis was restricted to ectomycorrhizal fungal (ECM) taxa (Rúa and Hoeksema, 2024). In New Zealand, introduced and consequently acclimatised landraces of *P. radiata* are considered a mixture of the mainland native populations from North America, i.e., Año Nuevo and Monterey, with some contributions from Cambria and the island populations of Cedros and Guadalupe (Burdon, 1997; Graham et al., 2022). Host ancestry, i.e., provenance, can influence the ECM communities that associate with young *P. radiata* roots (Rúa and Hoeksema, 2024), but whether this effect persists in older tree or trees outside their native regions is not known.

In this study, we investigated whether there was a host genetic basis for variation in the root microbiome in *Pinus radiata*. We analysed the bacterial and fungal root microbiomes of 132 genotypes of *P. radiata* from a nine-year-old clonal breeding trial. We tested the effects of host ancestry, which we inferred based on the genetic similarity to either the mainland or island native provenances of *P. radiata*, as well as host family and genotype on microbiome composition. We also determined whether the relative abundances of microbial taxa were sensitive to differences in host genetics and thus considered “heritable”, and whether abundance variations for these taxa were due to additive or non-additive host genetic effects. This study tests the importance of host genotype and ancestry in shaping microbiome composition and evaluates the potential of host breeding to modify the relative abundance of microbial taxa. We also provide critical insights that extend to other tree species where breeding programmes might seek to include microbiome-related traits to enhance climate or disease resistance.

## Materials and methods

### Trial information and selection of host genotypes

We sampled roots of *Pinus radiata* D.Don from clonal breeding trial BC52_1, established in 2013 by the Radiata Pine Breeding Company (RPBC). The trial is located in Kinleith Forest in the central North Island of New Zealand (38.370617° S, 175.929380° E), with soil classified as andisol (USDA-type classification) or well-draining pumice (S-map; New Zealand soil classification) (Lilburne et al., 2012). This trial comprised 650 unique genotypes, each with five clonal replicates (i.e., ramets) vegetatively propagated via cuttings. Genotypes were randomized across 100 blocks of 36 trees each throughout the 4.5 ha trial, with each block containing 36 different genotypes (i.e., single-tree incomplete block design, **Supplementary Figure 1**).

We selected three to six genotypes from each of 28 control-pollinated full-sib (i.e., both parents in common) families, for a total of 132 unique *P. radiata* genotypes that spanned the genetic diversity present in this trial (**Supplementary Table 1**). We sampled roots of four ramets of each genotype to give a total of 528 individual trees. Further details of the trial and selection process are provided in **Supplementary Methods**.

### Sample collections, processing and DNA extractions

Sampling was conducted over four weeks from mid-November 2022 using standardised *P. radiata* root microbiome collection methods (Sarah L. Addison et al., 2023). In brief, we collected two samples approximately 1 m from each side of a selected tree, maximising distance from adjacent trees. We removed non-target plants and the O-horizon, then dug holes approximately 10 cm x 10 cm x 10 cm using a clean spade, disinfected with ethanol wipes. Soil and roots from each of the two holes per tree were combined into a single sample in a new zip-lock bag, using fresh pairs of gloves for each tree. Samples were placed in insulated containers until arrival at the laboratory, then stored at 4 °C until processed within one week. Samples were thoroughly mixed before separating roots ≤ 3 mm in diameter and storing these at -20 °C for DNA extraction.

Prior to DNA extraction, roots were washed in 0.2 mM CaCl_2_ to remove adhering soil, then thoroughly chopped into approximately 2 mm segments and mixed. Approximately 100 mg of tissue was placed into Powerbead tubes (DNeasy PowerSoil Pro kit, Qiagen, Germany), homogenized using a Bead Ruptor Homogenizer (Omni International, USA), and DNA extracted in duplicate using Qiagen’s DNeasy PowerSoil Pro kit. Fluorometric quantification of DNA concentrations was determined using Quant-iT PicoGreen dsDNA reagents (Thermo Fisher Scientific, USA). Duplicate root DNA extractions were pooled in equimolar proportions to create 10 ng µl^-1^ working stocks for library preparation.

### Soil physicochemical properties

We calculated soil gravimetric moisture content by weighing subsamples before and after drying overnight at 65 °C. Soil pH, total carbon, and total nitrogen were predicted using diffuse reflectance mid-infrared spectroscopy (DRIFTS; Garrett et al., 2022). Where measurements were missing (moisture: n = 11; DRIFTS predictions: n = 1), samples were assigned the overall mean value. Variables were z-score standardised prior to principal component analysis, from which the first axis was extracted and used in all subsequent modelling steps, hereafter referred to as “soil PC1”. Further details are provided in **Supplementary Methods**.

### Microbiome library preparation and sequencing

Sequencing of bacterial and fungal microbiomes from root DNA followed methods described by the Earth Microbiome Programme (Thompson et al., 2017). We amplified the bacterial V4-V5 hypervariable region of 16S ribosomal RNA genes DNA with primers 515F and 806R (Parada et al., 2016); the 515F primer included additional sequencing adaptors and unique barcodes to enable sample pooling post-amplification. Fungal internal transcribed spacers (ITS) were amplified with primers ITS1F (Gardes and Bruns, 1993) and ITS2 (White et al., 1990); the ITS2 primer included additional sequencing adaptors and unique barcodes to enable sample pooling post-amplification. Libraries were sequenced using the MiSeq platform (Illumina, San Diego, CA, USA) at the Australian Genome Research Facility (AGRF, Melbourne, Australia). Bacterial 16S libraries were sequenced using 2 x 250 bp chemistry, and fungal ITS sequenced using 2 x 300 bp chemistry.

### Bioinformatics

Bacterial and fungal datasets were processed independently. Unless indicated, we used default filtering and trimming settings to process sequencing reads in the Dada2 pipeline (Callahan et al., 2016) in R version 4.4.0 (R Core Team, 2022). Primer sequences were removed from bacterial 16S sequences by truncation (truncLen = c(240,220)). As fungal ITS amplicons can vary in length, primers were removed from fungal ITS sequences using Cutadapt v4.4 (Martin, 2011). Dereplication of samples and merging of paired reads was performed prior to removal of chimeric sequences. Bacterial 16S and fungal ITS sequences are available in the sequence read archive under BioProject PRJNA1211146.

Taxonomy was assigned to amplicon sequence variants (ASVs) using the Ribosomal Database Project (RDP) training set v18 release 11.5 for bacteria (Wang et al., 2007), and the UNITE database v8.3 for fungi (Abarenkov et al., 2024). We removed ASVs if unclassified at the kingdom or phylum level, or identified as chloroplast, mitochondria, or archaea. Sequencing singletons were also removed. Samples were randomly rarefied without replacement to 8,063 (bacterial) or 10,718 (fungal) reads using ‘rrarefy’ in the vegan package (Oksanen et al., 2022). The rarefied datasets were used in all subsequent statistical analyses unless otherwise indicated.

### Statistical analyses

Visualisation of the relative abundances of different bacterial and fungal classes was conducted using Phyloseq (McMurdie and Holmes, 2013) and microviz (Barnett et al., 2021) packages in R. We defined the core bacterial and fungal microbiomes as ASVs present in at least 80% of samples that passed filtering. Bacterial and fungal ASV counts were square-root transformed to down-weight the influence of highly abundant ASVs.

Interpolation-based approaches including splines have been used to improve soil microbial diversity modelling in other studies (Constancias et al., 2015; Griffiths et al., 2011; Tedersoo et al., 2014). As accurate predictions of genetic signals can be impeded by spatial variability within a trial (Robbins et al., 2012), we accounted for spatial effects among samples by fitting a two dimensional spline (Robbins et al., 2012). This was performed using the package fields (Nychka et al., 2017) to fit a smooth surface to the transformed ASV counts using the coordinates of each tree within the trial. Variation that was not explained by the spline model was captured in the resulting residuals and represents the spatially-corrected ASV counts. As many of the subsequent analyses required positive values, we adjusted the residuals to be non-zero by adding the absolute of the minimum residual value for a given ASV to all the residual values for that ASV. These adjusted non-zero residuals were used in all downstream analyses on root ASV abundances, unless otherwise specified.

Beta diversity (community composition) was calculated using Bray-Curtis similarity (Bray and Curtis, 1957). As differences in beta diversity can reflect increased heterogeneity rather than shifts in mean composition, we first confirmed homogeneity of multivariate dispersion by comparing the distances to the centroid for host ancestry, family, and genotype using Permdisp performed in Primer7/Permanova+ using 999 permutations (Anderson, 2008; Clarke and Gorley, 2015; Clarke and Warwick, 2001). We considered a threshold of α = 0.05 for statistical significance in all analyses.

### Did composition of the root microbiome vary with host ancestry or family?

Ancestry of host genotypes was assigned based on overall genetic similarity to mainland (Cambria, Año Nuevo, and Cambria) or island (Cedros or Guadalupe Islands) populations of *P. radiata* (further details provided in **Supplementary Methods**). Most genotypes (n = 87) were more closely related to mainland populations, with only seven having island ancestry and 38 having mixed ancestry (**Supplementary Table 1**). Of the 28 host families, 18 consisted entirely of progeny with mainland ancestry, and four consisted of progeny with mixed ancestry. Six host families were classified as ambiguous with respect to ancestry: three families comprised both mainland and island progeny, and three comprised both mainland and mixed ancestry progeny.

We tested whether host ancestry was associated with composition of the root microbiome using PERMANOVA in Primer7/Permanova+, with ancestry as a fixed effect, host family as a random effect nested in ancestry, and host genotype as a random effect nested in host family. Sequencing batch and soil PC1 were fitted first in the model as fixed effects, with permutation of residuals under a reduced model with Type I sum of squares. Only host families with unambiguous ancestry assignments were included in the analysis (22 families, n = 102 genotypes, 389 samples for bacteria, 388 samples for fungi). This was repeated with all host families but excluded the ancestry term (n = 132 genotypes, 505 samples for bacteria; n = 130 genotypes, 500 samples for fungi).

### Were there heritable microbial taxa in the core microbiome and among host families?

Genetic parameters were estimated for each ASV under a general linear mixed model by residual maximum likelihood in ASReml-R (Butler et al., 2009). For each ASV, we modelled the unadjusted residuals (i.e., including negative values) after fitting a 2D thin plane spline to the square-root transformed counts (see above). Because we had both clonal replication of host genotypes and full-sib families in our study, we were able to include three host genetic terms in our model: an additive genetic term, a host family genetic term which captures 25% of the variance due to dominance, and a host genotype non-additive term which captures the remaining 75% of dominance variance and 100% of epistatic effects (Falconer, 1989). This was modelled as follows:


$$y=Xb+Z_{a}u_{a}+Z_{f}u_{f}+Z_{c}u_{c}+e$$

where y is the vector of ASV residuals per sample; b is the vector of fixed effects including intercept, sequencing batch and soil PC1 with the associated design matrix X**;** $u_{a}$, $u_{f}$, and $u_{c}$ are the vectors of random additive genetic effects, host family genetic effects, and host genotype non-additive genetic effects, respectively, with associated design matrices $Z_{a}$, $Z_{f}$, and $Z_{c}$**;** and e is the vector of residuals. Random effects and model residuals are normally distributed following $u_{a}\sim \;N(0,A\sigma_{a}^{2})$, $u_{f}\sim \;N(0,I\sigma_{f}^{2})$, $u_{c}\sim \;N(0,I\sigma_{c}^{2})$, and $e\;\sim \;N(0,I\sigma_{e}^{2})$, where I is the identity matrix, A is the pedigree-based relationship matrix among genotypes, and $\sigma_{a}^{2}$, $\sigma_{f}^{2}$, $\sigma_{c}^{2}$, and $\sigma_{e}^{2}$ are the additive genetic variance, host family genetic variance, genotype non-additive genetic variance, and residual variance, respectively.

Broad-sense (H^2^) and narrow-sense (h^2^) heritability were calculated as follows:


$$H^{2}\;=\frac{\left(\sigma_{a}^{2}+\sigma_{f}^{2}+\sigma_{c}^{2}\right)}{\left(\sigma_{a}^{2}+\sigma_{f}^{2}+\sigma_{c}^{2}+\sigma_{e}^{2}\right)}$$

$$h^{2}\;=\frac{\left(\sigma_{a}^{2}\right)}{\left(\sigma_{a}^{2}+\sigma_{f}^{2}+\sigma_{c}^{2}+\sigma_{e}^{2}\right)}$$

Significance of heritability estimates was determined by z-scores > |1.65|, which corresponds to α of 0.05. Phylograms showing relationships among core and heritable microbial taxa were generated in FastTree v2.1.11 (Price et al., 2010) using global pairwise alignments of ASV FASTA sequences (Mafft v7.526) (Katoh and Standley, 2013). Phylograms were midpoint-rooted in Mega v11.0.13 (Tamura et al., 2021) and annotated using iTOL version 7.0 (Letunic and Bork, 2024).

## Results

### Overview of soil physicochemical predictions

Mean soil moisture was 43.9% ± 6.0% (SD), ranging from 26% to 66% (**Supplementary Table 1**). Soil pH varied by more than a unit across the site, ranging from 4.15 to 5.24, with a mean of 4.63 ± 0.18 (SD). Mean total carbon and total nitrogen were 9.79% ± 2.46% (SD) and 0.45% ± 0.1% (SD), respectively. There were strong correlations between soil pH and total carbon (Pearson’s *r* = -0.78, *p* < 0.001), soil pH and total nitrogen (Pearson’s *r* = -0.70, *p* < 0.001), and total carbon and total nitrogen (Pearson’s *r* = 0.89, *p* < 0.001) (**Supplementary Figure 3**). Soil PC1 explained 78.47% of variation among the soil samples, with weighting of the individual soil variables as follows:


soil PC1=0.48(pH)−0.45(moisture)−0.54(total carbon)−0.52 (total nitrogen).

### The root microbiome of *Pinus radiata*

**Figure 1:**
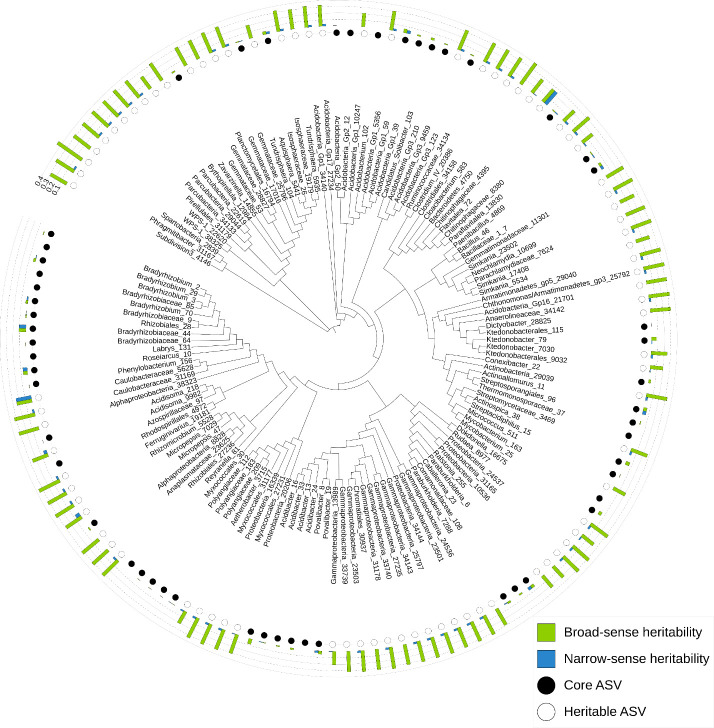
Phylogram showing relationships among the core (present in ≥ 80% of samples) and heritable (H^2^ > 0.2) ASVs from the bacterial root microbiomes of *Pinus radiata*. Three to six *P. radiata* progeny were sampled from each of 28 full-sib families, for a total of 132 unique host genotypes and 528 total trees. ASV genera or the next highest taxonomic assignment are indicated, followed by the ASV number. Full taxonomic assignments are available in **Supplementary Tables 3**, **7**.

Sequencing across all 528 samples yielded 28,205,889 raw bacterial paired-end reads. After processing through the Dada2 pipeline, filtering and rarefying, 4,071,815 bacterial reads remained across 505 samples. A total of 15,187 unique bacterial ASVs were defined of which *Alphaproteobacteria*, *Actinobacteria*, and *Gammaproteobacteria* were the most abundant classes (**Supplementary Figure 4a**). The most abundant bacterial ASV was identified as a *Bradyrhizobium* species. The core bacterial microbiome comprised 53 bacterial ASVs (0.36% of the total number of bacterial ASVs), which represented on average 35% of the reads per sample (**Supplementary Table 3**; **Figure 1**).

**Figure 2:**
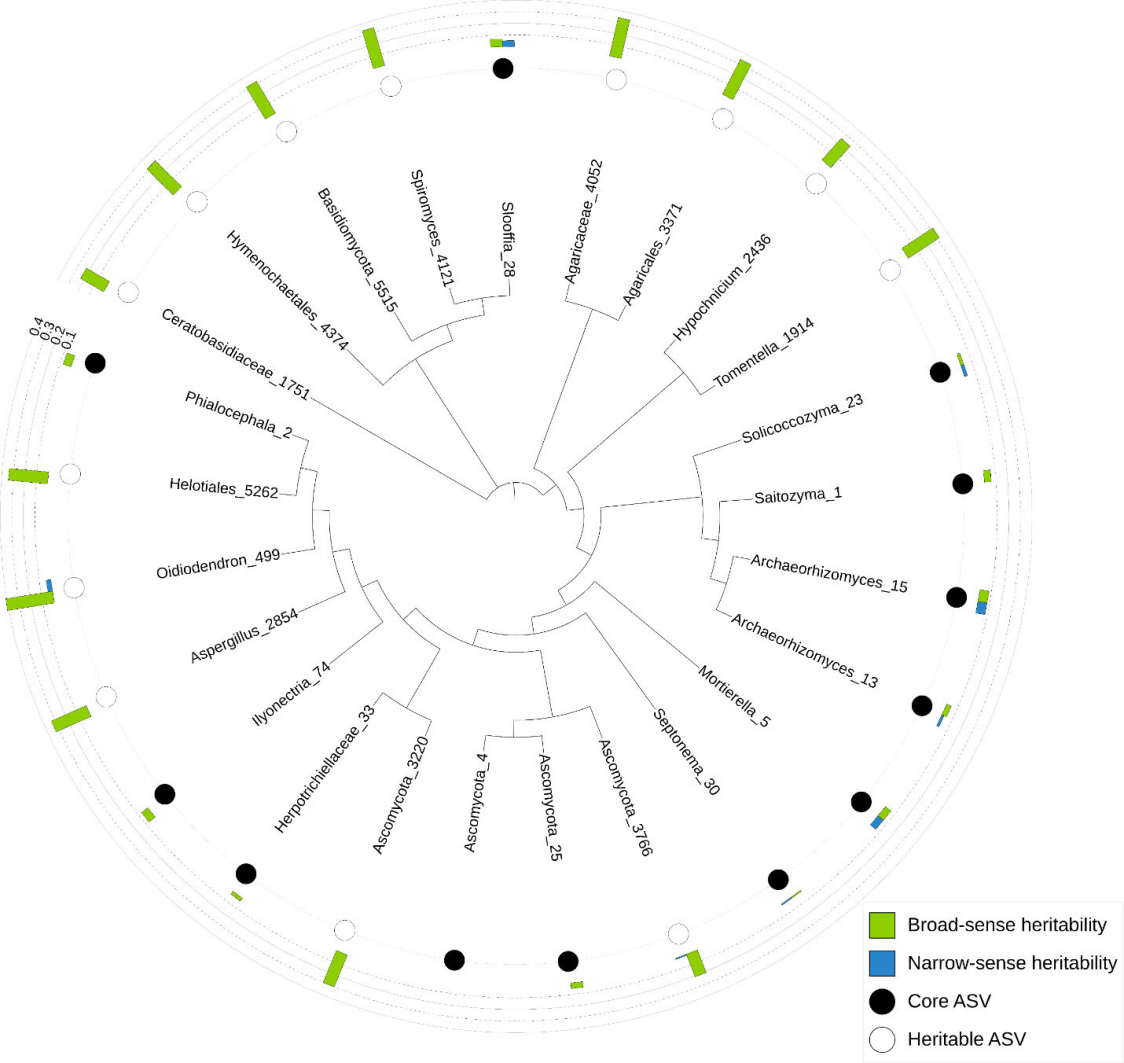
Phylogram showing relationships among the core (present in ≥ 80% of samples) and heritable (H^2^ > 0.2) ASVs from the fungal root microbiomes of *Pinus radiata*. Three to six *P. radiata* progeny were sampled from each of 28 full-sib families, for a total of 132 unique host genotypes and 528 total trees. ASV genera or the next highest taxonomic assignment are indicated, followed by the ASV number. Full taxonomic assignments are available in **Supplementary Tables 4**, **8**.

Sequencing yielded 31,723,317 raw fungal paired-end reads, which reduced to 5,359,000 fungal reads across 500 samples after processing through the Dada2 pipeline, filtering and rarefying. For fungi, 2,441 unique ASVs were defined of which *Agaricomycetes*, *Leotiomycetes* and an unclassified *Ascomycota* were the most abundant classes (**Supplementary Figure 4b**). The most abundant fungal ASV most closely matched *Phialocephala fortini.* The core fungal microbiome comprised 12 fungal ASVs (0.49% of the total number of fungal ASVs), which represented on average 25% of the reads per sample (**Supplementary Table 4**; **Figure 2**).

After spatial corrections, multivariate dispersion for both bacterial and fungal communities was homogenous for all factors included in the models: host ancestry, host family, host genotype, and sequencing batch (**Supplementary Table 5**).

### Composition of the root microbiome varied with host family

Host ancestry was not significant for either bacterial or fungal root microbiome composition and was excluded from further analyses (**Supplementary Table 6**). PERMANOVA testing revealed a significant association between host family and fungal but not bacterial root microbiome composition (**Table 1**). Individual genotype within family was not significant for the composition of either microbial community. Soil PC1 and sequencing batch had significant effects on both bacterial and fungal root microbiome composition (**Table 1**). Host family explained 8.0% of fungal microbiome composition variation.

**Table 1:** Summary PERMANOVA table showing the effect of host family (random effect) and host genotype (random effect, nested in family), fitted after covariates of sequencing batch and soil PC1 on spatially-corrected ASV counts for bacterial and fungal root microbiomes across 28 full-sib families of 132 Pinus radiata genotypes.

Term	Bacteria (n = 505)		Fungi (n = 500)	
	√CV^a^	*p*_perm_ ^b^	√CV^a^	*p*_perm_ ^b^
Sequencing batch	1.8	**0.001**	2.32	**0.001**
Soil PC1^c^	3.85	**0.001**	2.62	**0.001**
Family	1.02	0.178	1.74	**0.011**
Genotype (Family)	-1.41	0.798	1.28	0.246
Residual	21.13		21.16	

a √CV is the square root of the component of variation associated with each term. Negative variance components can result from how variance components are estimated (Anderson, 2008).

b *p***_perm_** is the probability statistic derived from 999 permutations of residuals under a reduced model.

c First principal component coordinates from principal component analysis of soil pH, gravimetric moisture, total carbon and total nitrogen.

Significant values (*p*_perm_ < 0.05) are bolded. Type I (sequential) sum of squares.

### The relative abundance of several non-core microbial taxa was heritable

**Figure 3:**
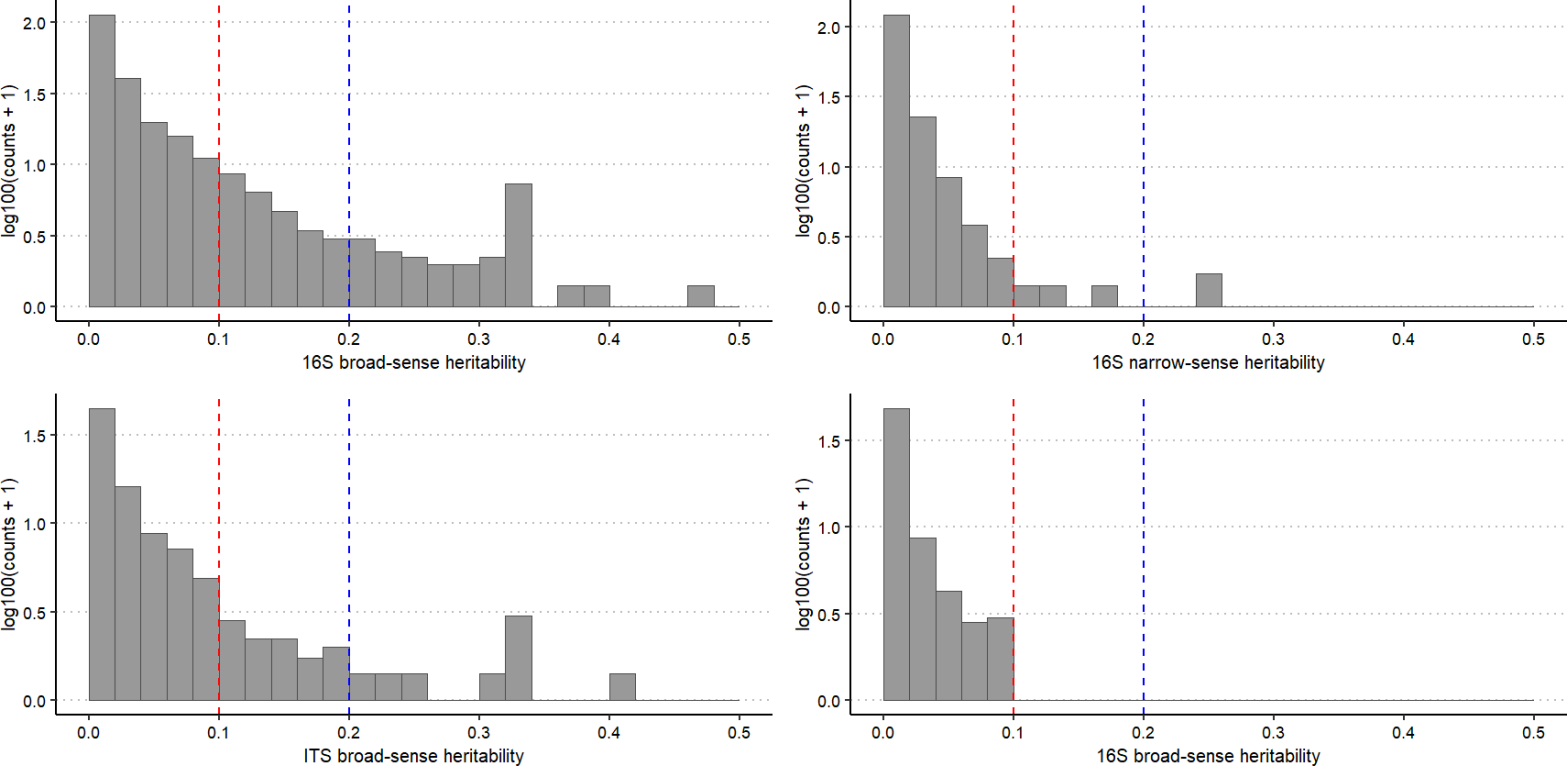
Histogram of the transformed (log100) counts of broad-sense heritability (H2) and narrow-sense heritability (h2) estimations for all bacterial (16S) and fungal (ITS) ASVs isolated from the root microbiomes of 132 genotypes of *Pinus radiata* from 28 full-sib families. Red lines indicate heritability of 0.1 and blue lines indicate heritability of 0.2.

There were 1,026 bacterial and 171 fungal ASVs with significant broad-sense heritability (α = 0.05), and 7,621 bacterial and 1,195 fungal ASVs with significant narrow-sense heritability. However, mean heritability for these ASVs was very low for both broad-sense (0.063 bacterial; 0.064 fungal) and narrow-sense heritability (0.0002 bacterial; 0.00005 fungal). The majority of bacterial and fungal ASVs had negligible (< 0.1) broad- and narrow-sense heritability (**Figure 3**). Mean heritability of the core bacterial microbiome was 0.02 (H^2^) and 0.01 (h^2^) (**Supplementary Table 3**), and 0.05 (H^2^) and 0.02 (h^2^) for the core fungal microbiome (**Supplementary Table 4**); most were not significant. The most heritable ASV in the core bacterial microbiome was from the family *Bradyrhizobiaceae* (0.11 for both H^2^ and h^2^) (**Supplementary Table 3**, **Figure 1**), and in the core fungal microbiome it was *Archaeorhizomyces* sp. (0.08 for both H^2^ and h^2^) (**Supplementary Table 4**; **Figure 2**).

There were 83 non-core bacterial ASVs with moderate broad-sense heritability (H^2^ > 0.2), of which two bacterial ASVs also had moderate narrow-sense heritability (h^2^ > 0.2; **Supplementary Table 7**; **Figure 1**): one from the *Chitinophagaceae* family and one from the *Caulobacteraceae* family. The bacterial genus with the highest heritability (H^2^ = 0.46) was identified as from the *Spartobacteria* class. There were 13 fungal ASVs with moderate broad-sense heritability (H^2^ > 0.2), but none with narrow-sense heritability > 0.04 (**Supplementary Table 8**; **Figure 2**). The fungal ASV with highest heritability (H^2^ = 0.41) was most closely matched to *Oidiodendron maius*.

## Discussion

We have provided evidence for host genetic influences on the bacterial and fungal root microbiomes of *Pinus radiata.* Using *P. radiata* as a model conifer system and sampling over 500 trees spanning 28 families and 132 unique genotypes, we demonstrate that host family was important for the overall composition of the fungal but not the bacterial root microbiome. However, this effect was not evident within host families where host genetic differences were perhaps too small to detect in our study. Within the bacterial and fungal microbiomes, we identified individual ASVs with moderate broad-sense heritability, two of which also had moderate narrow-sense heritability. These results indicate that complex genetic interactions in the host were important when considering variation in the abundance of these ASVs. This represents an important step forward in understanding how tree-microbe interactions are affected by host genetic effects, beyond tightly controlled glasshouse or common garden settings, and also in trees that are well beyond the juvenile stage of development. Confirmation of host genetic effects in shaping tree root microbiomes in a field setting could be useful for breeding programmes looking to select for microbiome-related traits, although this is severely limited by the inherently low to moderate narrow-sense heritability of most microbial ASVs.

### Whole root microbiome as a single trait

In the first part of our analysis, we considered the entire root-associated microbial community (bacterial or fungal) as a single host trait and tested whether the composition thereof varied with host ancestry, family, or genotype. We found that fungal communities showed significant host genetic effects at the family level, whereas bacterial communities did not. The lack of a host ancestry effect in our study could be due to limited representation of island genotypes in our study (7/132), or the result of domestication after multiple cycles of breeding in New Zealand. This contrasts with the germplasm used by Rúa and Hoeksema (2024) that was sourced more recently from native ranges, where a provenance effect was observed. Within-family effects were weak, with influences such as dispersal limitation, priority effects, and ecological drift, in conjunction with environmental factors such as soil pH, moisture and nutrient availability expected to dominate patterns of community assembly, making host genetic effects on the whole microbiome very difficult to detect. This compares favourably with other studies that suggest that the influence of site, time and developmental stage can mask plant genotype effects (Chen et al., 2019; Quiza et al., 2023; Sutherland et al., 2025; Veach et al., 2019).

The finding that fungal communities exhibited a detectable host genetic signal in an uncontrolled forest tree field-trial setting is unprecedented. It suggests that in *P. radiata*, root-associated fungal communities are more strongly and consistently shaped by host genetic background than bacteria, perhaps reflecting deeper evolutionary integration between conifers and their fungal partners (Rúa and Hoeksema, 2024). This points to the potential for fungal communities, considered at the whole-community scale, to be a genuine host-influenced trait. Other studies have similarly reported host effects as more important for fungal microbiome composition (Bergelson et al., 2019; Cregger et al., 2021; Zeng et al., 2022), while environmental conditions were more important for bacterial microbiome composition (Bahram et al., 2022; He et al., 2024). For bacteria, however, the absence of a clear host genetic signal at the whole-community level may simply reflect the sheer complexity and diversity of bacterial communities. Treating the entire bacterial community as a single trait is a crude approach: the aggregate signal can easily mask responses of specific bacterial groups or taxa that are indeed host-influenced. Detecting these finer-scale patterns requires moving beyond whole-community analysis to examine individual taxa or functional guilds.

### Heritability of taxa within the root microbiome

Narrowing the focus to individual microbial taxa revealed clearer host genetic influences on the root microbiome. Within the bacterial and fungal microbiomes, we identified several ASVs with moderate broad-sense heritability, indicating that both additive and non-additive host genetic effects influenced the relative abundance of these taxa. Two of these ASVs also had moderate narrow-sense heritability. The generally low to moderate heritability we observed is consistent with findings across other plants (Boyle et al., 2024; e.g., Deng et al., 2021; He et al., 2024; Lamit et al., 2016; Schweitzer et al., 2008; Sutherland et al., 2022; Wagner et al., 2016; Wallace et al., 2018; Walters et al., 2018) and animals (e.g., Grieneisen et al., 2021; Hess et al., 2023; Kurilshikov et al., 2021; Rothschild et al., 2018), where the heritability of microbial taxa is common but rarely strong. Importantly, our results demonstrate the importance of host genetic effects particularly in relation to the abundance of non-core bacterial and fungal taxa in the root microbiome.

The low heritability of the core root microbiome ASVs identified in this study was similar to a study in *Populus deltoides* where host genetic effects were less for abundant bacterial taxa (Yan et al., 2024). The core microbiome may consist of microbial taxa essential for host health. Low heritability for core taxa in our study indicates that variations in abundances were almost entirely due to environmental influences, which could indicate that host traits associated with maintaining these ASVs are conserved in the population under study. Alternatively, these core taxa may simply represent abundant generalists present in our study site. Our findings should, therefore, be compared with studies in other *P. radiata* populations to determine whether the “core” microbiome is indeed conserved across a broader genetic and environmental range.

Selecting for microbial taxa with low narrow-sense heritability will pose significant challenges for traditional breeding, as additive genetic variance is what drives the response to selection. If microbial taxa are shown to associate with host phenotypes of interest to the breeder, non-additive gene effects could be influenced through mate selection strategies (Mishra et al., 2017; Varona et al., 2018; Voss-Fels et al., 2019) where breeding programmes are based on control-pollination, however, this would still be challenging to implement. Other opportunities for genetic gains could also be achieved through clonal deployment strategies (Li and Dungey, 2018). The predominance of non-additive effects in our study supports results in other systems. For example, studies in switchgrass (Edwards et al., 2023) and maize (Wagner et al., 2020) have shown that non-additive genetic effects, including dominance and epistasis, strongly influence microbial associations (sometimes more so than additive effects). This complexity may reflect the involvement of multiple host traits such as root architecture (Maitra et al., 2024; Prada-Salcedo et al., 2021; Saleem et al., 2018; Szoboszlay et al., 2015), exudate chemistry, or immune responses (Alves et al., 2012; Burkhart et al., 2007; Vandenkoornhuyse et al., 2015), that interact in idiosyncratic ways to shape microbial colonisation.

The only other heritability study to our knowledge in the root microbiome of *P. radiata* was conducted in young seedlings (n = 472) and focused on specific ECM (Rúa and Hoeksema, 2024). While all 66 ECM fungal taxa under investigation were considered heritable, host pedigree relationships were not specified, and it is unclear whether the heritability reported included or excluded non-additive genetic effects. In contrast, while our study comprised a similar number of samples as Rúa and Hoeksema (2024), the pedigrees of our control-pollinated host families were three to four generations deep, allowing robust estimates of the relationships among host genotypes. Along with the inclusion of ramets and full-sib families in our study design, this enabled partitioning of non-additive genetic variance associated with host family and genotype from additive genetic variance and allowed us to estimate both broad- and narrow-sense heritability for ASVs. Our study also went well beyond ECM and quantified 15,187 bacterial and 2,441 fungal ASVs in 9-year post-establishment trees, making our study the first comprehensive evaluation of heritability of the root microbiome of *P. radiata* in more mature trees.

Some of the heritable taxa in our study have been previously reported in association with other plants, and in some cases also reported as heritable. For example, members of the *Chitinophagaceae* family, which contains several genera of gram-negative bacteria (Rosenberg, 2014), have been described from rhizospheric soil of an unspecified *Pinus* sp. (Huq et al., 2021) and *Pinus radiata* (Addison et al., 2021), and as endophytes of *Pinus pinaster* bark and sapwood (Proença et al., 2014). This bacterial family has also been isolated from *Pinus sylvestris* and *Pinus ponderosa* soils (Hartmann et al., 2023; Moore et al., 2023) and members are known to be enriched in lignin samples where they have a putative role in lignin and cellulose degradation (Hartmann et al., 2023; Wilhelm, 2018). Both the *Chitinophagaceae* and C*aulobacteraceae* families also had moderate narrow-sense heritability, and have been reported as heritable in the rhizosphere of *Poaceae* species including switchgrass (Sutherland et al., 2022), sorghum (Deng et al., 2021) and maize (He et al., 2024; Walters et al., 2018). The fungal ASV with the highest heritability was a match to *Oidiodendron maius*, which primarily forms ericoid mycorrhizas but can adopt an endophytic or saprotrophic lifestyle with non-ericaceous hosts (Mikheev et al., 2023). It has been found on the roots of *Pinus squamata* (Li and Sun, 2023), *Pinus sylvestris* (Sietiö et al., 2018), *Picea abies* and *Fagus sylvatica* (Qian et al., 1998), and *Pinus radiata* (Addison et al., 2018), and associated with differences in trunk straightness in *Pinus massoniana* (Li et al., 2023). It reportedly altered root morphology in blueberry plants (Baba et al., 2021) and improved growth in *Abies koreana* seedlings (Park et al., 2024). Beyond these limited studies, the potential impact of these microbial taxa on the broader microbiome composition or *P. radiata* fitness outcomes are unknown, and further studies are required.

## Conclusion

In conclusion, we detected minor but statistically significant host family effects for the root fungal microbiome of 9-year post-establishment *Pinus radiata* trees. Our study is also the first comprehensive evaluation of heritability of the root microbiome of *P. radiata*. Using a quantitative genetics approach, we have identified bacterial and fungal ASVs with relative abundances that are low to moderate heritability, although this did not apply to core microbial taxa and was predominantly explained by non-additive host genetic effects. This demonstration of heritability of root microbiome taxa provides the first indication that tree breeding programmes could select for microbiome-related traits, however, low narrow-sense heritabilities will limit their potential usefulness as selection traits in operational breeding programmes. We recommend follow-up studies in environments with increased stresses that might prompt stronger plant-microbe associations and in host populations with different gene frequencies that might reveal stronger host genetic effects.

## Data Availability

Sequences for 16S and ITS libraries are available in the Short Read Archive under BioProject PRJNA1211146. Additional information is available in **Supplementary Materials**.
