# Supplementary material for: Heritability of the Pinus radiata root microbiome
Source: Front Plant Sci. 2026 Apr 2;17:1793374. doi: 10.3389/fpls.2026.1793374 (PMC13083209; doi:10.3389/fpls.2026.1793374)
Supplement: Supplementary file 1 [file DataSheet1.docx]

### Supplementary methods

Trial information and selection of host genotypes

We sampled roots of *Pinus radiata* D.Don from clonal test trial BC52_1, established in 2013 by the Radiata Pine Breeding Company (RPBC) in Kinleith Forest in the central North Island of New Zealand. This trial comprised a total of 650 unique genotypes, each with five clonal replicates (i.e., ramets) vegetatively propagated via cuttings. These genotypes were generated through control-pollinated crosses among 55 progeny-tested parents, resulting in 63 different full-sib families (i.e., progeny shared both parents). Genotypes were randomized along with seedling controls and planted in incomplete blocks in a single-tree plot design, i.e., ramets of each genotype were distributed throughout the 4.5 ha trial, with each block containing a mixture of 36 different genotypes and controls (Figure S1).

We selected three to six genotypes from each of 28 control-pollinated full-sib families, for a total of 132 unique *P. radiata* genotypes (Table S1). Families were selected that spanned the genetic diversity in the trial based on historical genotyping (Graham et al., 2022), and contained at least three progeny with four or more surviving ramets. We excluded families with any previously-identified pedigree inconsistencies. As we wanted to test for host ancestry effects, we included as many families as possible with island or mixed ancestry (see “Assigning Ancestry” below). This was to address the uneven representation of the mainland vs island provenances of *P. radiata* in New Zealand, with most germplasm more closely related to the mainland provenances (Burdon et al., 1997).

Soil chemical properties

Gravimetric moisture content of the soil was calculated by weighing subsamples before and after drying overnight at 65°C. Soil chemical properties (pH, total carbon, total nitrogen) were predicted using diffuse reflectance mid-infrared spectroscopy (DRIFTS; Garrett et al., 2022). In brief, soil was air-dried and sieved to <2 mm, whereafter a 10 g subsample was ground for 180 s in a 45 ml zirconia ceramic grinding vial containing two 12.7 mm zirconia ceramic balls using a Spex800D mixer mill (Cole-Parmer, Metuchen, NJ, USA). Spectra were measured using an Invenio-S Fourier transform infrared spectrometer (Bruker, Billerica, Massachusetts, USA) with a Bruker HTS-XT (High Throughput Screening Extension) microplate reader fitted with a liquid nitrogen-cooled mercury cadmium telluride detector (Garrett et al., 2021). Four technical replicates were tested and the average spectrum used for chemical predictions against the USDA National Soil Survey Center–Kellogg Soil Survey Laboratory (NSSC KSSL) MIR spectral library (Safanelli et al., 2023; Sanderman et al., 2020).

To validate the predictions for chemical properties, 53 samples were selected for empirical laboratory analysis after performing principal component analysis (PCA) on the averaged spectra. Spectra were first truncated to 600-4000 wavenumbers and baseline transformed, and the PCA scores used in conditioned Latin hypercube sampling (cLHS) to select 53 samples (Figure S2) for traditional laboratory analysis at the University of Waikato’s ICP-MS Suite. Total carbon and total nitrogen were measured using a LECO FPS-21000 CNS thermal combustion furnace (modified Dumas), and pH measured in a 1:2.5 soil/water suspension. After comparing predicted values from the spectra and observed values from the traditional laboratory analyses, total carbon and pH were considered well predicted, evidenced by Lin’s concordance correlation coefficient values of 0.93 and 0.86 and R^2^ values of 0.90 and 0.82, respectively (Table S2). Total nitrogen was deemed adequately predicted with a Lin’s concordance correlation coefficient value of 0.55 and an R^2^ value of 0.87. Where measurements were missing (n = 11 for moisture; n = 1 for DRIFTS predictions), samples were assigned the overall mean for the affected variable. Variables were z-score standardised and used to perform a PCA, from which the first principal component axis was extracted and used in all subsequent modelling steps, hereafter referred to as soil PC1, where:

$$soil PC1=0.48\left( pH \right)-0.45\left( moisture \right)-0.54\left( total carbon \right)-0.52 \left( total nitrogen \right).$$

Assigning ancestry

Ancestry of the genotypes was inferred using historical single nucleotide polymorphism (SNP) profiles. A previous PCA of over 8,000 SNP profiles, which included our selected genotypes alongside representatives of ancestral populations, showed that variation along the first principal component correlated with ancestry, with genotypes from Guadalupe and Cedros Island populations having more negative values and those from the mainland populations of Año Nuevo, Monterey and Cambria having more positive values (Graham et al., 2022). As such, we used the position of our genotypes on the first PCA axis relative to the ancestral populations in the aforementioned PCA-based projection to assign putative ancestry as follows: “mainland” for genotypes more closely related to populations from Año Nuevo, Monterey and Cambria (PC1 > -0.01) ; “island” for genotypes more closely related to Guadalupe and Cedros Island populations (PC1 < -0.035); or “mixed” for genotypes that were related to both mainland and island populations (-0.03 < PC1 < -0.01).

***Microbiome library preparation – PCR conditions and clean-up***

Using TaKaRa’s Ex Taq Hot Start polymerase (Takara Bio, Kusatsu, Japan), amplification cycling conditions were as follows: for bacterial 16S, 94°C for 3 min; 35 cycles of 94°C for 40 s, 50°C for 60 s, and 72°C for 90 s, and a final extension at 72°C for 10 min; for fungal ITS, 94°C for 3 min; 35 cycles of 94°C for 30 s, 52°C for 30 s, and 72°C for 30 s, and a final extension at 72°C for 10 min. Amplification products were purified and standardised using SequalPrep 96-well Normalization Plates (Thermo Fisher Scientific), and pooled in batches of 176 to create a total of six libraries (three each for bacterial 16S and fungal ITS).

### Supplementary tables

**Table S1** Family and assigned ancestry information of 132 Pinus radiata genotypes (n = 528) across 28 full-sib families and three ancestry groups. Block indicates the location within the trial site (see Figure S1). Ancestry was assigned as either mainland, island, or mixed based on historical SNP profiles compared with those of representatives of ancestral populations. Soil pH, total carbon, and total nitrogen were predicted from diffuse reflectance mid-infrared spectra (DRIFTS). Samples with missing soil data, indicated by asterisk, were assigned the overall mean (in bold font).

| **Sample** | **Host genotype** | **Block** | **Parent 1^†^** | **Parent 2^†^** | **Family** | **Ancestry** | **Soil gravimetric moisture %** | **Soil pH** | **Soil total carbon %** | **Soil total nitrogen %** |
| --- | --- | --- | --- | --- | --- | --- | --- | --- | --- | --- |
| 1003_11_27 | 1003 | 11 | P1 | P20 | 10 | mainland | 0.43 | 4.58 | 11.15 | 0.55 |
| 1003_32_15 | 1003 | 32 | P1 | P20 | 10 | mainland | 0.40 | 4.51 | 8.81 | 0.47 |
| 1003_49_29 | 1003 | 49 | P1 | P20 | 10 | mainland | 0.47 | 4.44 | 9.90 | 0.46 |
| 1003_74_26 | 1003 | 74 | P1 | P20 | 10 | mainland | 0.48 | 4.74 | 8.94 | 0.41 |
| 1004_100_32 | 1004 | 100 | P1 | P20 | 10 | mainland | 0.56 | 4.28 | 12.53 | 0.58 |
| 1004_23_4 | 1004 | 23 | P1 | P20 | 10 | mainland | 0.33 | 4.82 | 7.31 | 0.39 |
| 1004_54_10 | 1004 | 54 | P1 | P20 | 10 | mainland | 0.42 | 5.00 | 7.45 | 0.37 |
| 1004_72_4 | 1004 | 72 | P1 | P20 | 10 | mainland | 0.45 | 5.02 | 5.55 | 0.29 |
| 1011_35_1 | 1011 | 35 | P1 | P20 | 10 | mainland | 0.55 | 4.54 | 11.49 | 0.49 |
| 1011_56_28 | 1011 | 56 | P1 | P20 | 10 | mainland | 0.42 | 4.76 | 7.75 | 0.40 |
| 1011_62_12 | 1011 | 62 | P1 | P20 | 10 | mainland | 0.40 | 4.71 | 7.09 | 0.30 |
| 1011_96_30 | 1011 | 96 | P1 | P20 | 10 | mainland | 0.51 | 4.39 | 14.42 | 0.62 |
| 1022_5_31 | 1022 | 5 | P1 | P20 | 10 | mainland | 0.37 | 4.71 | 8.08 | 0.38 |
| 1022_51_18 | 1022 | 51 | P1 | P20 | 10 | mainland | 0.47 | 4.63 | 8.46 | 0.40 |
| 1022_63_10 | 1022 | 63 | P1 | P20 | 10 | mainland | 0.35 | 4.59 | 10.08 | 0.47 |
| 1022_87_5 | 1022 | 87 | P1 | P20 | 10 | mainland | 0.43 | 4.58 | 11.48 | 0.57 |
| 1028_40_15 | 1028 | 40 | P1 | P20 | 10 | mainland | 0.47 | 4.51 | 10.30 | 0.52 |
| 1028_50_21 | 1028 | 50 | P1 | P20 | 10 | mainland | 0.39 | 4.61 | 9.07 | 0.48 |
| 1028_7_30 | 1028 | 7 | P1 | P20 | 10 | mainland | 0.45 | 4.62 | 9.23 | 0.46 |
| 1028_78_9 | 1028 | 78 | P1 | P20 | 10 | mainland | 0.41 | 4.71 | 9.27 | 0.31 |
| 1204_3_19 | 1204 | 3 | P2 | P21 | 12 | island | 0.42 | 4.64 | 9.69 | 0.44 |
| 1204_32_5 | 1204 | 32 | P2 | P21 | 12 | island | 0.49 | 4.38 | 11.55 | 0.53 |
| 1204_58_5 | 1204 | 58 | P2 | P21 | 12 | island | 0.53 | 4.34 | 15.19 | 0.61 |
| 1204_79_23 | 1204 | 79 | P2 | P21 | 12 | island | 0.54 | 4.46 | 15.35 | 0.62 |
| 1209_19_36 | 1209 | 19 | P2 | P21 | 12 | mixed | 0.44 | 4.73 | 10.64 | 0.51 |
| 1209_23_34 | 1209 | 23 | P2 | P21 | 12 | mixed | 0.51 | 4.60 | 8.07 | 0.40 |
| 1209_45_18 | 1209 | 45 | P2 | P21 | 12 | mixed | 0.39 | 4.73 | 9.17 | 0.40 |
| 1209_69_32 | 1209 | 69 | P2 | P21 | 12 | mixed | 0.42 | 4.75 | 8.69 | 0.37 |
| 1217_23_32 | 1217 | 23 | P2 | P21 | 12 | mixed | 0.39 | 4.44 | 9.32 | 0.47 |
| 1217_56_23* | 1217 | 56 | P2 | P21 | 12 | mixed | **0.44** | 4.73 | 8.69 | 0.42 |
| 1217_7_27 | 1217 | 7 | P2 | P21 | 12 | mixed | 0.49 | 4.62 | 9.34 | 0.47 |
| 1217_84_11 | 1217 | 84 | P2 | P21 | 12 | mixed | 0.47 | 4.83 | 8.88 | 0.44 |
| 1220_20_9 | 1220 | 20 | P2 | P21 | 12 | island | 0.45 | 4.71 | 10.54 | 0.56 |
| 1220_27_12 | 1220 | 27 | P2 | P21 | 12 | island | 0.48 | 4.52 | 11.46 | 0.57 |
| 1220_44_5 | 1220 | 44 | P2 | P21 | 12 | island | 0.46 | 4.64 | 7.38 | 0.39 |
| 1220_77_15 | 1220 | 77 | P2 | P21 | 12 | island | 0.44 | 4.63 | 8.64 | 0.44 |
| 1228_1_32 | 1228 | 1 | P2 | P21 | 12 | island | 0.52 | 4.52 | 11.53 | 0.55 |
| 1228_40_16 | 1228 | 40 | P2 | P21 | 12 | island | 0.50 | 4.38 | 11.60 | 0.55 |
| 1228_50_14 | 1228 | 50 | P2 | P21 | 12 | island | 0.47 | 4.53 | 15.07 | 0.61 |
| 1228_67_19 | 1228 | 67 | P2 | P21 | 12 | island | 0.47 | 4.54 | 10.83 | 0.47 |
| 1408_17_3 | 1408 | 17 | P3 | P22 | 14 | mainland | 0.50 | 4.63 | 11.49 | 0.51 |
| 1408_33_1 | 1408 | 33 | P3 | P22 | 14 | mainland | 0.36 | 4.75 | 8.11 | 0.38 |
| 1408_58_8 | 1408 | 58 | P3 | P22 | 14 | mainland | 0.52 | 4.42 | 12.51 | 0.56 |
| 1408_78_4 | 1408 | 78 | P3 | P22 | 14 | mainland | 0.40 | 4.85 | 8.15 | 0.38 |
| 1410_1_35 | 1410 | 1 | P3 | P22 | 14 | mainland | 0.56 | 4.46 | 13.91 | 0.59 |
| 1410_22_12 | 1410 | 22 | P3 | P22 | 14 | mainland | 0.36 | 5.17 | 3.17 | 0.15 |
| 1410_52_32 | 1410 | 52 | P3 | P22 | 14 | mainland | 0.45 | 4.48 | 9.83 | 0.47 |
| 1410_62_11 | 1410 | 62 | P3 | P22 | 14 | mainland | 0.48 | 4.47 | 11.74 | 0.48 |
| 1414_19_29 | 1414 | 19 | P3 | P22 | 14 | mainland | 0.44 | 5.01 | 7.46 | 0.38 |
| 1414_29_12 | 1414 | 29 | P3 | P22 | 14 | mainland | 0.43 | 4.83 | 8.08 | 0.34 |
| 1414_53_27 | 1414 | 53 | P3 | P22 | 14 | mainland | 0.49 | 4.66 | 9.86 | 0.49 |
| 1414_61_4 | 1414 | 61 | P3 | P22 | 14 | mainland | 0.37 | 4.71 | 9.25 | 0.48 |
| 1425_41_15 | 1425 | 41 | P3 | P22 | 14 | mainland | 0.51 | 4.42 | 12.87 | 0.50 |
| 1425_67_3 | 1425 | 67 | P3 | P22 | 14 | mainland | 0.38 | 4.80 | 8.23 | 0.34 |
| 1425_8_18 | 1425 | 8 | P3 | P22 | 14 | mainland | 0.40 | 4.71 | 10.36 | 0.53 |
| 1425_81_20 | 1425 | 81 | P3 | P22 | 14 | mainland | 0.41 | 4.92 | 7.18 | 0.38 |
| 1702_29_15 | 1702 | 29 | P4 | P23 | 17 | mainland | 0.43 | 4.65 | 9.87 | 0.47 |
| 1702_4_24 | 1702 | 4 | P4 | P23 | 17 | mainland | 0.36 | 4.64 | 6.23 | 0.33 |
| 1702_44_13 | 1702 | 44 | P4 | P23 | 17 | mainland | 0.41 | 4.69 | 8.47 | 0.36 |
| 1702_63_34 | 1702 | 63 | P4 | P23 | 17 | mainland | 0.42 | 4.62 | 11.74 | 0.56 |
| 1703_10_31* | 1703 | 10 | P4 | P23 | 17 | mainland | **0.44** | 4.85 | 9.63 | 0.48 |
| 1703_34_7 | 1703 | 34 | P4 | P23 | 17 | mainland | 0.44 | 4.73 | 9.33 | 0.44 |
| 1703_49_11 | 1703 | 49 | P4 | P23 | 17 | mainland | 0.45 | 4.46 | 14.17 | 0.59 |
| 1703_71_24 | 1703 | 71 | P4 | P23 | 17 | mainland | 0.51 | 4.61 | 10.26 | 0.55 |
| 1718_26_35 | 1718 | 26 | P4 | P23 | 17 | mainland | 0.47 | 4.36 | 17.28 | 0.65 |
| 1718_58_21 | 1718 | 58 | P4 | P23 | 17 | mainland | 0.42 | 4.75 | 8.84 | 0.40 |
| 1718_61_12 | 1718 | 61 | P4 | P23 | 17 | mainland | 0.41 | 4.71 | 7.96 | 0.36 |
| 1718_8_32 | 1718 | 8 | P4 | P23 | 17 | mainland | 0.41 | 4.85 | 7.97 | 0.39 |
| 1727_18_29 | 1727 | 18 | P4 | P23 | 17 | mainland | 0.46 | 4.50 | 10.85 | 0.57 |
| 1727_40_5 | 1727 | 40 | P4 | P23 | 17 | mainland | 0.44 | 4.70 | 8.95 | 0.48 |
| 1727_53_3 | 1727 | 53 | P4 | P23 | 17 | mainland | 0.43 | 4.69 | 8.65 | 0.40 |
| 1727_77_8 | 1727 | 77 | P4 | P23 | 17 | mainland | 0.45 | 4.59 | 9.61 | 0.43 |
| 2108_36_17 | 2108 | 36 | P5 | P24 | 21 | mainland | 0.43 | 4.94 | 7.84 | 0.39 |
| 2108_4_6 | 2108 | 4 | P5 | P24 | 21 | mainland | 0.34 | 5.13 | 4.65 | 0.22 |
| 2108_43_20 | 2108 | 43 | P5 | P24 | 21 | mainland | 0.38 | 4.78 | 7.74 | 0.40 |
| 2108_77_19 | 2108 | 77 | P5 | P24 | 21 | mainland | 0.43 | 4.65 | 8.30 | 0.41 |
| 2116_17_5 | 2116 | 17 | P5 | P24 | 21 | mainland | 0.43 | 4.83 | 8.07 | 0.38 |
| 2116_28_9 | 2116 | 28 | P5 | P24 | 21 | mainland | 0.40 | 4.56 | 9.39 | 0.49 |
| 2116_51_15 | 2116 | 51 | P5 | P24 | 21 | mainland | 0.45 | 4.69 | 8.90 | 0.42 |
| 2116_62_10 | 2116 | 62 | P5 | P24 | 21 | mainland | 0.43 | 4.56 | 11.04 | 0.53 |
| 2121_39_18 | 2121 | 39 | P5 | P24 | 21 | mainland | 0.45 | 4.68 | 9.94 | 0.42 |
| 2121_4_9 | 2121 | 4 | P5 | P24 | 21 | mainland | 0.37 | 5.11 | 4.10 | 0.22 |
| 2121_57_6 | 2121 | 57 | P5 | P24 | 21 | mainland | 0.44 | 4.45 | 10.43 | 0.57 |
| 2121_66_36 | 2121 | 66 | P5 | P24 | 21 | mainland | 0.44 | 4.53 | 12.12 | 0.58 |
| 2123_20_10 | 2123 | 20 | P5 | P24 | 21 | mainland | 0.46 | 4.51 | 11.34 | 0.50 |
| 2123_27_6 | 2123 | 27 | P5 | P24 | 21 | mainland | 0.35 | 5.03 | 4.66 | 0.25 |
| 2123_51_27 | 2123 | 51 | P5 | P24 | 21 | mainland | 0.43 | 4.62 | 9.18 | 0.51 |
| 2123_61_3 | 2123 | 61 | P5 | P24 | 21 | mainland | 0.42 | 4.54 | 10.60 | 0.47 |
| 2301_38_3 | 2301 | 38 | P6 | P16 | 23 | mainland | 0.60 | 4.15 | 23.45 | 0.75 |
| 2301_5_35 | 2301 | 5 | P6 | P16 | 23 | mainland | 0.39 | 4.66 | 8.33 | 0.38 |
| 2301_56_7 | 2301 | 56 | P6 | P16 | 23 | mainland | 0.48 | 4.78 | 8.44 | 0.44 |
| 2301_69_18 | 2301 | 69 | P6 | P16 | 23 | mainland | 0.44 | 4.44 | 12.78 | 0.62 |
| 2302_40_19 | 2302 | 40 | P6 | P16 | 23 | mainland | 0.40 | 4.71 | 8.69 | 0.38 |
| 2302_46_8 | 2302 | 46 | P6 | P16 | 23 | mainland | 0.39 | 4.63 | 10.51 | 0.48 |
| 2302_6_6 | 2302 | 6 | P6 | P16 | 23 | mainland | 0.30 | 4.81 | 8.44 | 0.42 |
| 2302_68_32* | 2302 | 68 | P6 | P16 | 23 | mainland | 0.43 | **4.63** | **9.77** | **0.45** |
| 2305_3_34 | 2305 | 3 | P6 | P16 | 23 | mainland | 0.40 | 5.22 | 4.56 | 0.24 |
| 2305_37_12 | 2305 | 37 | P6 | P16 | 23 | mainland | 0.39 | 4.98 | 6.89 | 0.32 |
| 2305_45_17 | 2305 | 45 | P6 | P16 | 23 | mainland | 0.37 | 4.75 | 9.10 | 0.44 |
| 2305_76_30 | 2305 | 76 | P6 | P16 | 23 | mainland | 0.45 | 4.43 | 12.63 | 0.52 |
| 2307_12_13 | 2307 | 12 | P6 | P16 | 23 | mainland | 0.49 | 4.71 | 10.75 | 0.46 |
| 2307_33_35 | 2307 | 33 | P6 | P16 | 23 | mainland | 0.41 | 4.54 | 9.30 | 0.36 |
| 2307_55_9 | 2307 | 55 | P6 | P16 | 23 | mainland | 0.41 | 4.92 | 7.21 | 0.37 |
| 2307_74_24 | 2307 | 74 | P6 | P16 | 23 | mainland | 0.47 | 4.82 | 8.51 | 0.40 |
| 2316_20_28 | 2316 | 20 | P6 | P16 | 23 | mainland | 0.56 | 4.40 | 13.44 | 0.53 |
| 2316_38_26 | 2316 | 38 | P6 | P16 | 23 | mainland | 0.48 | 4.51 | 10.82 | 0.50 |
| 2316_59_12 | 2316 | 59 | P6 | P16 | 23 | mainland | 0.33 | 4.91 | 6.17 | 0.34 |
| 2316_66_14 | 2316 | 66 | P6 | P16 | 23 | mainland | 0.54 | 4.53 | 11.67 | 0.51 |
| 2603_10_27 | 2603 | 10 | P7 | P21 | 26 | island | 0.45 | 4.69 | 9.21 | 0.43 |
| 2603_40_13 | 2603 | 40 | P7 | P21 | 26 | island | 0.37 | 4.79 | 7.64 | 0.36 |
| 2603_58_25 | 2603 | 58 | P7 | P21 | 26 | island | 0.54 | 4.52 | 9.26 | 0.48 |
| 2603_74_2 | 2603 | 74 | P7 | P21 | 26 | island | 0.44 | 4.89 | 7.87 | 0.45 |
| 2611_12_14 | 2611 | 12 | P7 | P21 | 26 | mixed | 0.47 | 4.62 | 9.72 | 0.47 |
| 2611_28_18 | 2611 | 28 | P7 | P21 | 26 | mixed | 0.35 | 4.96 | 6.54 | 0.35 |
| 2611_52_19 | 2611 | 52 | P7 | P21 | 26 | mixed | 0.43 | 4.63 | 8.51 | 0.42 |
| 2611_67_7 | 2611 | 67 | P7 | P21 | 26 | mixed | 0.37 | 4.83 | 7.28 | 0.33 |
| 2613_12_11 | 2613 | 12 | P7 | P21 | 26 | island | 0.50 | 4.59 | 10.99 | 0.57 |
| 2613_30_35 | 2613 | 30 | P7 | P21 | 26 | island | 0.49 | 4.86 | 6.65 | 0.33 |
| 2613_58_13 | 2613 | 58 | P7 | P21 | 26 | island | 0.43 | 4.67 | 9.21 | 0.38 |
| 2613_68_7 | 2613 | 68 | P7 | P21 | 26 | island | 0.40 | 4.68 | 8.10 | 0.42 |
| 2616_19_11 | 2616 | 19 | P7 | P21 | 26 | island | 0.53 | 4.37 | 14.30 | 0.63 |
| 2616_32_13 | 2616 | 32 | P7 | P21 | 26 | island | 0.45 | 4.47 | 10.40 | 0.44 |
| 2616_54_28 | 2616 | 54 | P7 | P21 | 26 | island | 0.66 | 4.32 | 19.15 | 0.64 |
| 2616_63_6 | 2616 | 63 | P7 | P21 | 26 | island | 0.39 | 4.68 | 8.80 | 0.41 |
| 2629_39_20 | 2629 | 39 | P7 | P21 | 26 | mixed | 0.48 | 4.67 | 9.38 | 0.41 |
| 2629_41_25 | 2629 | 41 | P7 | P21 | 26 | mixed | 0.42 | 4.54 | 9.30 | 0.44 |
| 2629_79_24 | 2629 | 79 | P7 | P21 | 26 | mixed | 0.45 | 4.49 | 10.91 | 0.52 |
| 2629_98_18 | 2629 | 98 | P7 | P21 | 26 | mixed | 0.56 | 4.45 | 9.90 | 0.48 |
| 303_18_5 | 303 | 18 | P8 | P25 | 3 | mainland | 0.45 | 5.05 | 6.96 | 0.38 |
| 303_38_23 | 303 | 38 | P8 | P25 | 3 | mainland | 0.39 | 4.78 | 7.35 | 0.38 |
| 303_51_32 | 303 | 51 | P8 | P25 | 3 | mainland | 0.42 | 4.59 | 8.97 | 0.40 |
| 303_68_1 | 303 | 68 | P8 | P25 | 3 | mainland | 0.39 | 4.82 | 9.11 | 0.43 |
| 3103_24_2 | 3103 | 24 | P2 | P17 | 31 | mixed | 0.36 | 4.80 | 8.22 | 0.45 |
| 3103_49_1 | 3103 | 49 | P2 | P17 | 31 | mixed | 0.47 | 4.38 | 14.39 | 0.59 |
| 3103_61_16 | 3103 | 61 | P2 | P17 | 31 | mixed | 0.50 | 4.43 | 15.49 | 0.58 |
| 3103_9_19 | 3103 | 9 | P2 | P17 | 31 | mixed | 0.49 | 4.64 | 10.15 | 0.50 |
| 3110_27_23 | 3110 | 27 | P2 | P17 | 31 | mixed | 0.52 | 4.66 | 11.52 | 0.48 |
| 3110_3_3 | 3110 | 3 | P2 | P17 | 31 | mixed | 0.44 | 4.58 | 6.97 | 0.33 |
| 3110_50_26 | 3110 | 50 | P2 | P17 | 31 | mixed | 0.51 | 4.32 | 12.75 | 0.53 |
| 3110_74_15 | 3110 | 74 | P2 | P17 | 31 | mixed | 0.54 | 4.60 | 11.42 | 0.61 |
| 3112_26_13 | 3112 | 26 | P2 | P17 | 31 | mixed | 0.48 | 4.45 | 13.05 | 0.63 |
| 3112_3_12 | 3112 | 3 | P2 | P17 | 31 | mixed | 0.38 | 5.23 | 2.87 | 0.15 |
| 3112_52_16 | 3112 | 52 | P2 | P17 | 31 | mixed | 0.42 | 4.83 | 8.26 | 0.44 |
| 3112_77_28 | 3112 | 77 | P2 | P17 | 31 | mixed | 0.45 | 4.47 | 11.27 | 0.50 |
| 3114_15_25 | 3114 | 15 | P2 | P17 | 31 | island | 0.38 | 4.32 | 8.89 | 0.31 |
| 3114_24_1 | 3114 | 24 | P2 | P17 | 31 | island | 0.36 | 4.67 | 10.50 | 0.53 |
| 3114_59_27 | 3114 | 59 | P2 | P17 | 31 | island | 0.42 | 4.53 | 11.99 | 0.56 |
| 3114_74_27 | 3114 | 74 | P2 | P17 | 31 | island | 0.47 | 4.78 | 8.88 | 0.40 |
| 3117_2_32 | 3117 | 2 | P2 | P17 | 31 | mixed | 0.37 | 4.64 | 6.80 | 0.29 |
| 3117_23_20 | 3117 | 23 | P2 | P17 | 31 | mixed | 0.35 | 4.55 | 7.94 | 0.34 |
| 3117_44_35 | 3117 | 44 | P2 | P17 | 31 | mixed | 0.42 | 4.58 | 9.20 | 0.44 |
| 3117_73_28 | 3117 | 73 | P2 | P17 | 31 | mixed | 0.55 | 4.57 | 11.44 | 0.56 |
| 3123_2_10 | 3123 | 2 | P2 | P17 | 31 | mixed | 0.48 | 4.55 | 9.56 | 0.46 |
| 3123_30_14 | 3123 | 30 | P2 | P17 | 31 | mixed | 0.39 | 5.00 | 7.10 | 0.37 |
| 3123_49_21 | 3123 | 49 | P2 | P17 | 31 | mixed | 0.47 | 4.47 | 11.24 | 0.43 |
| 3123_67_12 | 3123 | 67 | P2 | P17 | 31 | mixed | 0.33 | 4.86 | 4.39 | 0.25 |
| 318_14_14 | 318 | 14 | P8 | P25 | 3 | mainland | 0.43 | 4.35 | 10.10 | 0.40 |
| 318_30_20 | 318 | 30 | P8 | P25 | 3 | mainland | 0.48 | 4.44 | 10.99 | 0.50 |
| 318_57_5 | 318 | 57 | P8 | P25 | 3 | mainland | 0.44 | 4.64 | 8.38 | 0.40 |
| 318_71_20 | 318 | 71 | P8 | P25 | 3 | mainland | 0.65 | 4.19 | 20.53 | 0.74 |
| 321_28_21 | 321 | 28 | P8 | P25 | 3 | mainland | 0.35 | 4.78 | 7.72 | 0.34 |
| 321_43_32 | 321 | 43 | P8 | P25 | 3 | mainland | 0.43 | 4.75 | 7.86 | 0.39 |
| 321_73_9 | 321 | 73 | P8 | P25 | 3 | mainland | 0.57 | 4.58 | 10.95 | 0.45 |
| 321_8_19 | 321 | 8 | P8 | P25 | 3 | mainland | 0.44 | 4.48 | 11.98 | 0.49 |
| 323_23_25 | 323 | 23 | P8 | P25 | 3 | mainland | 0.42 | 4.63 | 9.20 | 0.48 |
| 323_4_13 | 323 | 4 | P8 | P25 | 3 | mainland | 0.32 | 4.76 | 5.66 | 0.29 |
| 323_55_13 | 323 | 55 | P8 | P25 | 3 | mainland | 0.38 | 4.91 | 8.27 | 0.47 |
| 323_65_14 | 323 | 65 | P8 | P25 | 3 | mainland | 0.43 | 4.80 | 8.87 | 0.45 |
| 3304_35_34 | 3304 | 35 | P9 | P2 | 33 | mixed | 0.44 | 4.48 | 9.69 | 0.47 |
| 3304_44_16 | 3304 | 44 | P9 | P2 | 33 | mixed | 0.44 | 4.60 | 9.03 | 0.44 |
| 3304_5_29 | 3304 | 5 | P9 | P2 | 33 | mixed | 0.34 | 4.72 | 7.91 | 0.32 |
| 3304_69_36 | 3304 | 69 | P9 | P2 | 33 | mixed | 0.42 | 4.85 | 7.81 | 0.44 |
| 3307_14_26 | 3307 | 14 | P9 | P2 | 33 | mixed | 0.39 | 4.80 | 8.72 | 0.43 |
| 3307_30_18 | 3307 | 30 | P9 | P2 | 33 | mixed | 0.46 | 4.65 | 9.06 | 0.46 |
| 3307_45_4 | 3307 | 45 | P9 | P2 | 33 | mixed | 0.46 | 4.63 | 9.08 | 0.42 |
| 3307_67_8 | 3307 | 67 | P9 | P2 | 33 | mixed | 0.40 | 4.80 | 8.16 | 0.32 |
| 3316_13_14 | 3316 | 13 | P9 | P2 | 33 | mixed | 0.43 | 4.45 | 13.19 | 0.62 |
| 3316_36_7 | 3316 | 36 | P9 | P2 | 33 | mixed | 0.34 | 5.10 | 5.34 | 0.28 |
| 3316_51_2 | 3316 | 51 | P9 | P2 | 33 | mixed | 0.43 | 4.69 | 9.36 | 0.40 |
| 3316_80_16 | 3316 | 80 | P9 | P2 | 33 | mixed | 0.48 | 4.46 | 10.39 | 0.51 |
| 3317_1_13 | 3317 | 1 | P9 | P2 | 33 | mixed | 0.47 | 4.65 | 9.04 | 0.48 |
| 3317_37_34 | 3317 | 37 | P9 | P2 | 33 | mixed | 0.44 | 4.44 | 9.93 | 0.46 |
| 3317_55_4 | 3317 | 55 | P9 | P2 | 33 | mixed | 0.45 | 4.87 | 8.79 | 0.39 |
| 3317_63_15 | 3317 | 63 | P9 | P2 | 33 | mixed | 0.41 | 4.61 | 8.99 | 0.48 |
| 3320_27_34 | 3320 | 27 | P9 | P2 | 33 | mixed | 0.40 | 5.13 | 3.94 | 0.20 |
| 3320_42_6 | 3320 | 42 | P9 | P2 | 33 | mixed | 0.49 | 4.51 | 13.22 | 0.52 |
| 3320_67_13 | 3320 | 67 | P9 | P2 | 33 | mixed | 0.42 | 4.39 | 12.12 | 0.62 |
| 3320_9_25 | 3320 | 9 | P9 | P2 | 33 | mixed | 0.46 | 4.76 | 8.22 | 0.40 |
| 3507_12_28 | 3507 | 12 | P10 | P11 | 35 | mainland | 0.49 | 4.44 | 12.22 | 0.56 |
| 3507_26_23 | 3507 | 26 | P10 | P11 | 35 | mainland | 0.38 | 4.79 | 8.10 | 0.41 |
| 3507_47_4 | 3507 | 47 | P10 | P11 | 35 | mainland | 0.46 | 4.29 | 11.69 | 0.57 |
| 3507_71_29 | 3507 | 71 | P10 | P11 | 35 | mainland | 0.48 | 4.63 | 9.02 | 0.36 |
| 3510_2_25 | 3510 | 2 | P10 | P11 | 35 | mainland | 0.39 | 4.73 | 7.01 | 0.36 |
| 3510_22_1 | 3510 | 22 | P10 | P11 | 35 | mainland | 0.37 | 4.65 | 8.24 | 0.39 |
| 3510_44_12 | 3510 | 44 | P10 | P11 | 35 | mainland | 0.45 | 4.37 | 11.82 | 0.54 |
| 3510_76_18 | 3510 | 76 | P10 | P11 | 35 | mainland | 0.48 | 4.65 | 13.29 | 0.54 |
| 3511_20_34 | 3511 | 20 | P10 | P11 | 35 | mainland | 0.50 | 4.60 | 10.46 | 0.43 |
| 3511_26_26 | 3511 | 26 | P10 | P11 | 35 | mainland | 0.47 | 4.50 | 11.23 | 0.45 |
| 3511_41_35 | 3511 | 41 | P10 | P11 | 35 | mainland | 0.43 | 4.55 | 8.94 | 0.43 |
| 3511_75_6 | 3511 | 75 | P10 | P11 | 35 | mainland | 0.44 | 4.65 | 10.48 | 0.52 |
| 3525_28_16 | 3525 | 28 | P10 | P11 | 35 | mainland | 0.37 | 4.82 | 8.26 | 0.44 |
| 3525_6_17 | 3525 | 6 | P10 | P11 | 35 | mainland | 0.41 | 4.44 | 12.54 | 0.59 |
| 3525_60_18 | 3525 | 60 | P10 | P11 | 35 | mainland | 0.45 | 4.39 | 12.08 | 0.59 |
| 3525_71_2 | 3525 | 71 | P10 | P11 | 35 | mainland | 0.50 | 4.53 | 10.82 | 0.54 |
| 3528_17_20 | 3528 | 17 | P10 | P11 | 35 | mainland | 0.43 | 4.61 | 8.78 | 0.42 |
| 3528_21_26 | 3528 | 21 | P10 | P11 | 35 | mainland | 0.42 | 4.63 | 8.72 | 0.34 |
| 3528_45_14 | 3528 | 45 | P10 | P11 | 35 | mainland | 0.45 | 4.77 | 7.19 | 0.37 |
| 3528_63_27 | 3528 | 63 | P10 | P11 | 35 | mainland | 0.35 | 4.58 | 9.79 | 0.44 |
| 3701_20_15 | 3701 | 20 | P6 | P26 | 37 | mainland | 0.50 | 4.60 | 15.16 | 0.75 |
| 3701_21_15 | 3701 | 21 | P6 | P26 | 37 | mainland | 0.45 | 4.78 | 10.05 | 0.45 |
| 3701_51_30 | 3701 | 51 | P6 | P26 | 37 | mainland | 0.38 | 4.85 | 7.30 | 0.37 |
| 3701_64_21 | 3701 | 64 | P6 | P26 | 37 | mainland | 0.40 | 4.69 | 9.35 | 0.40 |
| 3706_18_1 | 3706 | 18 | P6 | P26 | 37 | mainland | 0.42 | 4.95 | 7.41 | 0.34 |
| 3706_22_22 | 3706 | 22 | P6 | P26 | 37 | mainland | 0.36 | 4.68 | 7.72 | 0.30 |
| 3706_47_31 | 3706 | 47 | P6 | P26 | 37 | mainland | 0.43 | 4.56 | 10.63 | 0.54 |
| 3706_73_14 | 3706 | 73 | P6 | P26 | 37 | mainland | 0.48 | 4.46 | 11.58 | 0.62 |
| 3720_17_21 | 3720 | 17 | P6 | P26 | 37 | mainland | 0.31 | 4.79 | 5.95 | 0.29 |
| 3720_40_12 | 3720 | 40 | P6 | P26 | 37 | mainland | 0.43 | 4.74 | 10.06 | 0.46 |
| 3720_44_7 | 3720 | 44 | P6 | P26 | 37 | mainland | 0.43 | 4.43 | 10.70 | 0.48 |
| 3720_64_17 | 3720 | 64 | P6 | P26 | 37 | mainland | 0.32 | 4.82 | 6.98 | 0.31 |
| 3721_11_1 | 3721 | 11 | P6 | P26 | 37 | mainland | 0.48 | 4.45 | 10.85 | 0.48 |
| 3721_30_6 | 3721 | 30 | P6 | P26 | 37 | mainland | 0.61 | 4.31 | 12.85 | 0.51 |
| 3721_48_22 | 3721 | 48 | P6 | P26 | 37 | mainland | 0.46 | 4.43 | 11.91 | 0.55 |
| 3721_63_29 | 3721 | 63 | P6 | P26 | 37 | mainland | 0.35 | 4.77 | 8.43 | 0.36 |
| 3726_19_32 | 3726 | 19 | P6 | P26 | 37 | mainland | 0.47 | 4.54 | 11.01 | 0.48 |
| 3726_39_8 | 3726 | 39 | P6 | P26 | 37 | mainland | 0.55 | 4.44 | 12.25 | 0.59 |
| 3726_59_2 | 3726 | 59 | P6 | P26 | 37 | mainland | 0.37 | 4.68 | 9.16 | 0.42 |
| 3726_79_19 | 3726 | 79 | P6 | P26 | 37 | mainland | 0.42 | 4.49 | 11.15 | 0.53 |
| 4004_17_1 | 4004 | 17 | P11 | P21 | 40 | mixed | 0.43 | 4.81 | 8.61 | 0.34 |
| 4004_36_31 | 4004 | 36 | P11 | P21 | 40 | mixed | 0.49 | 4.81 | 8.08 | 0.40 |
| 4004_60_15 | 4004 | 60 | P11 | P21 | 40 | mixed | 0.40 | 4.66 | 8.52 | 0.41 |
| 4004_74_9 | 4004 | 74 | P11 | P21 | 40 | mixed | 0.51 | 4.69 | 9.69 | 0.52 |
| 4023_15_8 | 4023 | 15 | P11 | P21 | 40 | mainland | 0.31 | 5.24 | 3.79 | 0.20 |
| 4023_49_18 | 4023 | 49 | P11 | P21 | 40 | mainland | 0.46 | 4.33 | 11.49 | 0.48 |
| 4023_64_16 | 4023 | 64 | P11 | P21 | 40 | mainland | 0.35 | 4.76 | 8.27 | 0.44 |
| 4023_99_11 | 4023 | 99 | P11 | P21 | 40 | mainland | 0.51 | 4.42 | 11.61 | 0.55 |
| 4024_38_15 | 4024 | 38 | P11 | P21 | 40 | mainland | 0.45 | 4.69 | 8.36 | 0.41 |
| 4024_56_18 | 4024 | 56 | P11 | P21 | 40 | mainland | 0.49 | 4.67 | 8.89 | 0.43 |
| 4024_80_24 | 4024 | 80 | P11 | P21 | 40 | mainland | 0.46 | 4.69 | 8.30 | 0.43 |
| 4024_98_14 | 4024 | 98 | P11 | P21 | 40 | mainland | 0.51 | 4.34 | 12.93 | 0.60 |
| 4026_31_22 | 4026 | 31 | P11 | P21 | 40 | mainland | 0.34 | 4.67 | 8.37 | 0.40 |
| 4026_6_8 | 4026 | 6 | P11 | P21 | 40 | mainland | 0.37 | 4.68 | 8.75 | 0.42 |
| 4026_60_8 | 4026 | 60 | P11 | P21 | 40 | mainland | 0.37 | 4.62 | 9.78 | 0.50 |
| 4026_68_28 | 4026 | 68 | P11 | P21 | 40 | mainland | 0.36 | 4.96 | 7.25 | 0.34 |
| 4305_12_1 | 4305 | 12 | P12 | P27 | 43 | mainland | 0.46 | 4.72 | 10.85 | 0.57 |
| 4305_27_11 | 4305 | 27 | P12 | P27 | 43 | mainland | 0.40 | 4.68 | 8.78 | 0.44 |
| 4305_44_26 | 4305 | 44 | P12 | P27 | 43 | mainland | 0.43 | 4.58 | 10.71 | 0.52 |
| 4305_68_36 | 4305 | 68 | P12 | P27 | 43 | mainland | 0.42 | 4.54 | 9.95 | 0.43 |
| 4306_1_8 | 4306 | 1 | P12 | P27 | 43 | mainland | 0.48 | 4.48 | 10.32 | 0.54 |
| 4306_36_36 | 4306 | 36 | P12 | P27 | 43 | mainland | 0.41 | 4.84 | 8.01 | 0.37 |
| 4306_51_25 | 4306 | 51 | P12 | P27 | 43 | mainland | 0.44 | 4.60 | 9.41 | 0.48 |
| 4306_72_8 | 4306 | 72 | P12 | P27 | 43 | mainland | 0.47 | 4.58 | 10.16 | 0.53 |
| 4307_16_35 | 4307 | 16 | P12 | P27 | 43 | mainland | 0.40 | 4.82 | 6.44 | 0.31 |
| 4307_31_12 | 4307 | 31 | P12 | P27 | 43 | mainland | 0.53 | 4.45 | 10.23 | 0.45 |
| 4307_55_19 | 4307 | 55 | P12 | P27 | 43 | mainland | 0.49 | 4.51 | 10.50 | 0.50 |
| 4307_63_35 | 4307 | 63 | P12 | P27 | 43 | mainland | 0.46 | 4.70 | 10.22 | 0.47 |
| 4317_32_20 | 4317 | 32 | P12 | P27 | 43 | mainland | 0.40 | 4.68 | 8.55 | 0.40 |
| 4317_43_9 | 4317 | 43 | P12 | P27 | 43 | mainland | 0.44 | 4.60 | 8.92 | 0.37 |
| 4317_7_9 | 4317 | 7 | P12 | P27 | 43 | mainland | 0.50 | 4.60 | 12.39 | 0.58 |
| 4317_71_36 | 4317 | 71 | P12 | P27 | 43 | mainland | 0.47 | 4.60 | 8.78 | 0.43 |
| 4324_17_12 | 4324 | 17 | P12 | P27 | 43 | mainland | 0.50 | 4.34 | 15.16 | 0.66 |
| 4324_26_32 | 4324 | 26 | P12 | P27 | 43 | mainland | 0.42 | 4.68 | 6.20 | 0.36 |
| 4324_59_9 | 4324 | 59 | P12 | P27 | 43 | mainland | 0.36 | 4.64 | 8.70 | 0.46 |
| 4324_79_27 | 4324 | 79 | P12 | P27 | 43 | mainland | 0.47 | 4.47 | 12.61 | 0.55 |
| 4602_13_8 | 4602 | 13 | P13 | P28 | 46 | mixed | 0.39 | 4.63 | 10.35 | 0.49 |
| 4602_38_21 | 4602 | 38 | P13 | P28 | 46 | mixed | 0.55 | 4.35 | 14.73 | 0.62 |
| 4602_48_34 | 4602 | 48 | P13 | P28 | 46 | mixed | 0.50 | 4.53 | 9.72 | 0.43 |
| 4602_72_7 | 4602 | 72 | P13 | P28 | 46 | mixed | 0.55 | 4.46 | 15.51 | 0.65 |
| 4604_25_14 | 4604 | 25 | P13 | P28 | 46 | mixed | 0.50 | 4.62 | 10.18 | 0.40 |
| 4604_5_16 | 4604 | 5 | P13 | P28 | 46 | mixed | 0.39 | 4.57 | 7.66 | 0.32 |
| 4604_60_27 | 4604 | 60 | P13 | P28 | 46 | mixed | 0.43 | 4.55 | 10.37 | 0.50 |
| 4604_79_29 | 4604 | 79 | P13 | P28 | 46 | mixed | 0.43 | 4.59 | 11.14 | 0.59 |
| 4612_31_1 | 4612 | 31 | P13 | P28 | 46 | mixed | 0.35 | 4.64 | 8.20 | 0.43 |
| 4612_5_8 | 4612 | 5 | P13 | P28 | 46 | mixed | 0.38 | 4.50 | 8.03 | 0.37 |
| 4612_56_32 | 4612 | 56 | P13 | P28 | 46 | mixed | 0.51 | 4.42 | 11.79 | 0.53 |
| 4612_73_17 | 4612 | 73 | P13 | P28 | 46 | mixed | 0.47 | 4.74 | 9.03 | 0.46 |
| 4617_20_2 | 4617 | 20 | P13 | P28 | 46 | mixed | 0.40 | 4.63 | 9.33 | 0.39 |
| 4617_40_25 | 4617 | 40 | P13 | P28 | 46 | mixed | 0.34 | 4.84 | 7.09 | 0.33 |
| 4617_45_16 | 4617 | 45 | P13 | P28 | 46 | mixed | 0.40 | 4.71 | 9.01 | 0.43 |
| 4617_74_28 | 4617 | 74 | P13 | P28 | 46 | mixed | 0.53 | 4.64 | 12.67 | 0.56 |
| 4625_1_19 | 4625 | 1 | P13 | P28 | 46 | mainland | 0.56 | 4.47 | 16.08 | 0.72 |
| 4625_31_28 | 4625 | 31 | P13 | P28 | 46 | mainland | 0.27 | 4.71 | 6.91 | 0.38 |
| 4625_50_7 | 4625 | 50 | P13 | P28 | 46 | mainland | 0.41 | 4.85 | 7.18 | 0.37 |
| 4625_76_21 | 4625 | 76 | P13 | P28 | 46 | mainland | 0.44 | 4.40 | 11.79 | 0.58 |
| 4903_10_35 | 4903 | 10 | P14 | P1 | 49 | mainland | 0.45 | 4.70 | 9.75 | 0.43 |
| 4903_27_2 | 4903 | 27 | P14 | P1 | 49 | mainland | 0.54 | 4.27 | 15.78 | 0.60 |
| 4903_56_14 | 4903 | 56 | P14 | P1 | 49 | mainland | 0.48 | 4.70 | 9.73 | 0.47 |
| 4903_62_35 | 4903 | 62 | P14 | P1 | 49 | mainland | 0.39 | 4.58 | 9.03 | 0.47 |
| 4911_16_26 | 4911 | 16 | P14 | P1 | 49 | mainland | 0.45 | 4.71 | 8.19 | 0.45 |
| 4911_40_29 | 4911 | 40 | P14 | P1 | 49 | mainland | 0.39 | 4.80 | 7.74 | 0.42 |
| 4911_51_23 | 4911 | 51 | P14 | P1 | 49 | mainland | 0.50 | 4.33 | 12.01 | 0.35 |
| 4911_75_12 | 4911 | 75 | P14 | P1 | 49 | mainland | 0.46 | 4.68 | 10.88 | 0.55 |
| 4919_1_29 | 4919 | 1 | P14 | P1 | 49 | mainland | 0.51 | 4.40 | 11.44 | 0.59 |
| 4919_28_30 | 4919 | 28 | P14 | P1 | 49 | mainland | 0.52 | 4.29 | 11.59 | 0.48 |
| 4919_45_29 | 4919 | 45 | P14 | P1 | 49 | mainland | 0.39 | 4.69 | 9.68 | 0.45 |
| 4919_61_31 | 4919 | 61 | P14 | P1 | 49 | mainland | 0.48 | 4.43 | 13.00 | 0.59 |
| 4922_2_15 | 4922 | 2 | P14 | P1 | 49 | mainland | 0.47 | 4.78 | 9.62 | 0.42 |
| 4922_29_32 | 4922 | 29 | P14 | P1 | 49 | mainland | 0.44 | 4.74 | 9.60 | 0.49 |
| 4922_47_11 | 4922 | 47 | P14 | P1 | 49 | mainland | 0.40 | 4.70 | 7.57 | 0.38 |
| 4922_79_30 | 4922 | 79 | P14 | P1 | 49 | mainland | 0.47 | 4.40 | 13.33 | 0.57 |
| 4930_2_27 | 4930 | 2 | P14 | P1 | 49 | mainland | 0.44 | 4.43 | 8.84 | 0.37 |
| 4930_34_29 | 4930 | 34 | P14 | P1 | 49 | mainland | 0.46 | 4.66 | 9.53 | 0.44 |
| 4930_60_2 | 4930 | 60 | P14 | P1 | 49 | mainland | 0.51 | 4.32 | 14.43 | 0.58 |
| 4930_73_21 | 4930 | 73 | P14 | P1 | 49 | mainland | 0.54 | 4.44 | 11.92 | 0.56 |
| 5018_25_20 | 5018 | 25 | P9 | P23 | 50 | mainland | 0.43 | 4.67 | 9.89 | 0.48 |
| 5018_48_32* | 5018 | 48 | P9 | P23 | 50 | mainland | **0.44** | 4.48 | 10.68 | 0.44 |
| 5018_67_4 | 5018 | 67 | P9 | P23 | 50 | mainland | 0.41 | 4.58 | 9.21 | 0.44 |
| 5018_9_15* | 5018 | 9 | P9 | P23 | 50 | mainland | **0.44** | 4.37 | 14.88 | 0.62 |
| 5020_37_5 | 5020 | 37 | P9 | P23 | 50 | mainland | 0.39 | 4.76 | 8.11 | 0.36 |
| 5020_58_9 | 5020 | 58 | P9 | P23 | 50 | mainland | 0.50 | 4.60 | 10.35 | 0.52 |
| 5020_75_7 | 5020 | 75 | P9 | P23 | 50 | mainland | 0.39 | 4.64 | 9.70 | 0.51 |
| 5020_87_10 | 5020 | 87 | P9 | P23 | 50 | mainland | 0.40 | 4.97 | 9.86 | 0.61 |
| 5022_10_3 | 5022 | 10 | P9 | P23 | 50 | mainland | 0.44 | 4.65 | 9.02 | 0.39 |
| 5022_25_32 | 5022 | 25 | P9 | P23 | 50 | mainland | 0.38 | 4.91 | 7.00 | 0.35 |
| 5022_42_3 | 5022 | 42 | P9 | P23 | 50 | mainland | 0.34 | 4.69 | 8.07 | 0.39 |
| 5022_62_31 | 5022 | 62 | P9 | P23 | 50 | mainland | 0.40 | 4.57 | 10.70 | 0.48 |
| 5025_26_10 | 5025 | 26 | P9 | P23 | 50 | mainland | 0.44 | 4.60 | 9.05 | 0.44 |
| 5025_5_10 | 5025 | 5 | P9 | P23 | 50 | mainland | 0.37 | 4.55 | 6.22 | 0.31 |
| 5025_50_1 | 5025 | 50 | P9 | P23 | 50 | mainland | 0.40 | 4.94 | 6.25 | 0.33 |
| 5025_70_30 | 5025 | 70 | P9 | P23 | 50 | mainland | 0.43 | 4.57 | 9.42 | 0.47 |
| 513_18_6 | 513 | 18 | P15 | P25 | 5 | mainland | 0.44 | 5.01 | 6.92 | 0.37 |
| 513_40_30 | 513 | 40 | P15 | P25 | 5 | mainland | 0.40 | 4.74 | 8.44 | 0.45 |
| 513_41_22 | 513 | 41 | P15 | P25 | 5 | mainland | 0.46 | 4.60 | 10.48 | 0.50 |
| 513_88_23 | 513 | 88 | P15 | P25 | 5 | mainland | 0.43 | 4.45 | 10.24 | 0.50 |
| 516_16_25 | 516 | 16 | P15 | P25 | 5 | mainland | 0.41 | 4.79 | 8.58 | 0.41 |
| 516_30_12 | 516 | 30 | P15 | P25 | 5 | mainland | 0.41 | 4.72 | 8.82 | 0.38 |
| 516_47_8 | 516 | 47 | P15 | P25 | 5 | mainland | 0.49 | 4.35 | 12.12 | 0.44 |
| 516_78_23 | 516 | 78 | P15 | P25 | 5 | mainland | 0.47 | 4.61 | 9.51 | 0.34 |
| 518_1_10 | 518 | 1 | P15 | P25 | 5 | mainland | 0.50 | 4.62 | 10.20 | 0.53 |
| 518_39_13 | 518 | 39 | P15 | P25 | 5 | mainland | 0.44 | 4.63 | 8.86 | 0.31 |
| 518_49_4 | 518 | 49 | P15 | P25 | 5 | mainland | 0.40 | 4.52 | 9.37 | 0.48 |
| 518_88_22 | 518 | 88 | P15 | P25 | 5 | mainland | 0.43 | 4.55 | 8.67 | 0.39 |
| 5504_36_23 | 5504 | 36 | P16 | P3 | 55 | mainland | 0.38 | 4.32 | 15.85 | 0.65 |
| 5504_53_36 | 5504 | 53 | P16 | P3 | 55 | mainland | 0.47 | 4.64 | 10.57 | 0.54 |
| 5504_6_14 | 5504 | 6 | P16 | P3 | 55 | mainland | 0.41 | 4.73 | 9.44 | 0.43 |
| 5504_64_36 | 5504 | 64 | P16 | P3 | 55 | mainland | 0.40 | 4.68 | 10.37 | 0.46 |
| 5506_40_10 | 5506 | 40 | P16 | P3 | 55 | mainland | 0.49 | 4.51 | 11.92 | 0.58 |
| 5506_41_36 | 5506 | 41 | P16 | P3 | 55 | mainland | 0.40 | 4.65 | 7.64 | 0.31 |
| 5506_5_20 | 5506 | 5 | P16 | P3 | 55 | mainland | 0.35 | 4.66 | 8.29 | 0.39 |
| 5506_62_33 | 5506 | 62 | P16 | P3 | 55 | mainland | 0.40 | 4.73 | 8.76 | 0.45 |
| 5509_19_10 | 5509 | 19 | P16 | P3 | 55 | mainland | 0.44 | 4.71 | 8.03 | 0.42 |
| 5509_35_3 | 5509 | 35 | P16 | P3 | 55 | mainland | 0.44 | 4.60 | 8.27 | 0.37 |
| 5509_49_12 | 5509 | 49 | P16 | P3 | 55 | mainland | 0.48 | 4.35 | 15.52 | 0.58 |
| 5509_80_9 | 5509 | 80 | P16 | P3 | 55 | mainland | 0.46 | 4.45 | 10.19 | 0.50 |
| 5511_16_12 | 5511 | 16 | P16 | P3 | 55 | mainland | 0.42 | 4.72 | 8.47 | 0.38 |
| 5511_38_1 | 5511 | 38 | P16 | P3 | 55 | mainland | 0.42 | 4.83 | 8.14 | 0.42 |
| 5511_46_6 | 5511 | 46 | P16 | P3 | 55 | mainland | 0.40 | 4.54 | 9.99 | 0.47 |
| 5511_61_15 | 5511 | 61 | P16 | P3 | 55 | mainland | 0.47 | 4.50 | 10.28 | 0.47 |
| 5521_13_15 | 5521 | 13 | P16 | P3 | 55 | mainland | 0.39 | 4.67 | 9.66 | 0.44 |
| 5521_23_30 | 5521 | 23 | P16 | P3 | 55 | mainland | 0.43 | 4.65 | 9.29 | 0.46 |
| 5521_58_36 | 5521 | 58 | P16 | P3 | 55 | mainland | 0.38 | 4.64 | 8.68 | 0.39 |
| 5521_72_2* | 5521 | 72 | P16 | P3 | 55 | mainland | **0.44** | 4.99 | 6.53 | 0.35 |
| 5601_10_25 | 5601 | 10 | P17 | P1 | 56 | mixed | 0.49 | 4.61 | 10.77 | 0.56 |
| 5601_35_27 | 5601 | 35 | P17 | P1 | 56 | mixed | 0.52 | 4.33 | 16.72 | 0.73 |
| 5601_51_28 | 5601 | 51 | P17 | P1 | 56 | mixed | 0.41 | 4.64 | 7.71 | 0.40 |
| 5601_63_18 | 5601 | 63 | P17 | P1 | 56 | mixed | 0.37 | 4.59 | 10.36 | 0.49 |
| 5602_25_26 | 5602 | 25 | P17 | P1 | 56 | mixed | 0.49 | 4.49 | 10.22 | 0.46 |
| 5602_59_23 | 5602 | 59 | P17 | P1 | 56 | mixed | 0.39 | 4.68 | 9.33 | 0.47 |
| 5602_61_6 | 5602 | 61 | P17 | P1 | 56 | mixed | 0.41 | 4.47 | 10.89 | 0.48 |
| 5602_7_22 | 5602 | 7 | P17 | P1 | 56 | mixed | 0.49 | 4.63 | 10.50 | 0.52 |
| 5603_19_25 | 5603 | 19 | P17 | P1 | 56 | mixed | 0.36 | 4.88 | 7.28 | 0.35 |
| 5603_34_28 | 5603 | 34 | P17 | P1 | 56 | mixed | 0.39 | 4.73 | 7.65 | 0.34 |
| 5603_57_14 | 5603 | 57 | P17 | P1 | 56 | mixed | 0.45 | 4.54 | 10.59 | 0.56 |
| 5603_68_31 | 5603 | 68 | P17 | P1 | 56 | mixed | 0.38 | 4.72 | 7.82 | 0.39 |
| 5611_14_12 | 5611 | 14 | P17 | P1 | 56 | mixed | 0.34 | 4.79 | 6.32 | 0.30 |
| 5611_21_25 | 5611 | 21 | P17 | P1 | 56 | mixed | 0.52 | 4.39 | 13.74 | 0.50 |
| 5611_42_28 | 5611 | 42 | P17 | P1 | 56 | mixed | 0.44 | 4.60 | 10.04 | 0.48 |
| 5611_73_16 | 5611 | 73 | P17 | P1 | 56 | mixed | 0.52 | 4.68 | 9.88 | 0.47 |
| 5618_14_1 | 5618 | 14 | P17 | P1 | 56 | mixed | 0.41 | 4.51 | 9.51 | 0.41 |
| 5618_27_32 | 5618 | 27 | P17 | P1 | 56 | mixed | 0.45 | 4.59 | 9.37 | 0.33 |
| 5618_49_24 | 5618 | 49 | P17 | P1 | 56 | mixed | 0.38 | 4.56 | 10.21 | 0.44 |
| 5618_77_32 | 5618 | 77 | P17 | P1 | 56 | mixed | 0.44 | 4.56 | 9.72 | 0.44 |
| 5801_10_8 | 5801 | 10 | P13 | P29 | 58 | mainland | 0.47 | 4.71 | 9.30 | 0.40 |
| 5801_26_8 | 5801 | 26 | P13 | P29 | 58 | mainland | 0.43 | 4.56 | 10.00 | 0.52 |
| 5801_46_28 | 5801 | 46 | P13 | P29 | 58 | mainland | 0.30 | 4.72 | 7.36 | 0.32 |
| 5801_76_35 | 5801 | 76 | P13 | P29 | 58 | mainland | 0.47 | 4.52 | 10.96 | 0.46 |
| 5811_24_31 | 5811 | 24 | P13 | P29 | 58 | mixed | 0.37 | 4.81 | 9.72 | 0.48 |
| 5811_51_1 | 5811 | 51 | P13 | P29 | 58 | mixed | 0.43 | 4.76 | 8.46 | 0.44 |
| 5811_79_20 | 5811 | 79 | P13 | P29 | 58 | mixed | 0.41 | 4.74 | 10.08 | 0.47 |
| 5811_8_11 | 5811 | 8 | P13 | P29 | 58 | mixed | 0.53 | 4.61 | 11.14 | 0.53 |
| 5815_19_26 | 5815 | 19 | P13 | P29 | 58 | mixed | 0.40 | 4.87 | 8.35 | 0.35 |
| 5815_31_26 | 5815 | 31 | P13 | P29 | 58 | mixed | 0.26 | 4.87 | 5.51 | 0.28 |
| 5815_42_5 | 5815 | 42 | P13 | P29 | 58 | mixed | 0.37 | 4.78 | 8.60 | 0.41 |
| 5815_71_10 | 5815 | 71 | P13 | P29 | 58 | mixed | 0.63 | 4.23 | 20.96 | 0.72 |
| 5817_14_34 | 5817 | 14 | P13 | P29 | 58 | mixed | 0.38 | 4.75 | 9.27 | 0.39 |
| 5817_25_11 | 5817 | 25 | P13 | P29 | 58 | mixed | 0.43 | 4.92 | 7.15 | 0.36 |
| 5817_51_11 | 5817 | 51 | P13 | P29 | 58 | mixed | 0.45 | 4.78 | 8.21 | 0.43 |
| 5817_78_7 | 5817 | 78 | P13 | P29 | 58 | mixed | 0.40 | 4.69 | 8.55 | 0.40 |
| 5826_35_11 | 5826 | 35 | P13 | P29 | 58 | mixed | 0.42 | 4.66 | 8.73 | 0.40 |
| 5826_54_9 | 5826 | 54 | P13 | P29 | 58 | mixed | 0.47 | 4.84 | 9.78 | 0.43 |
| 5826_6_2 | 5826 | 6 | P13 | P29 | 58 | mixed | 0.36 | 4.70 | 9.11 | 0.42 |
| 5826_65_23 | 5826 | 65 | P13 | P29 | 58 | mixed | 0.43 | 4.66 | 9.90 | 0.52 |
| 5901_2_31 | 5901 | 2 | P18 | P22 | 59 | mainland | 0.47 | 4.48 | 9.01 | 0.43 |
| 5901_31_16 | 5901 | 31 | P18 | P22 | 59 | mainland | 0.37 | 4.74 | 9.44 | 0.45 |
| 5901_57_3 | 5901 | 57 | P18 | P22 | 59 | mainland | 0.54 | 4.26 | 14.05 | 0.71 |
| 5901_63_32 | 5901 | 63 | P18 | P22 | 59 | mainland | 0.38 | 4.65 | 9.05 | 0.40 |
| 5902_12_7 | 5902 | 12 | P18 | P22 | 59 | mainland | 0.56 | 4.51 | 12.68 | 0.58 |
| 5902_25_25 | 5902 | 25 | P18 | P22 | 59 | mainland | 0.50 | 4.56 | 9.85 | 0.37 |
| 5902_46_18 | 5902 | 46 | P18 | P22 | 59 | mainland | 0.39 | 4.55 | 10.00 | 0.43 |
| 5902_64_32 | 5902 | 64 | P18 | P22 | 59 | mainland | 0.37 | 4.77 | 10.20 | 0.49 |
| 5903_30_15 | 5903 | 30 | P18 | P22 | 59 | mainland | 0.45 | 4.69 | 9.23 | 0.43 |
| 5903_4_14 | 5903 | 4 | P18 | P22 | 59 | mainland | 0.30 | 4.46 | 6.81 | 0.28 |
| 5903_50_18 | 5903 | 50 | P18 | P22 | 59 | mainland | 0.44 | 4.63 | 9.32 | 0.37 |
| 5903_65_34 | 5903 | 65 | P18 | P22 | 59 | mainland | 0.35 | 4.68 | 8.44 | 0.44 |
| 5905_36_11 | 5905 | 36 | P18 | P22 | 59 | mainland | 0.36 | 4.71 | 6.10 | 0.33 |
| 5905_49_36 | 5905 | 49 | P18 | P22 | 59 | mainland | 0.52 | 4.65 | 7.73 | 0.34 |
| 5905_7_24 | 5905 | 7 | P18 | P22 | 59 | mainland | 0.44 | 4.72 | 8.96 | 0.40 |
| 5905_73_5 | 5905 | 73 | P18 | P22 | 59 | mainland | 0.48 | 5.01 | 7.32 | 0.35 |
| 5909_2_21 | 5909 | 2 | P18 | P22 | 59 | mainland | 0.58 | 4.33 | 12.12 | 0.54 |
| 5909_22_11 | 5909 | 22 | P18 | P22 | 59 | mainland | 0.30 | 5.00 | 4.40 | 0.23 |
| 5909_41_23 | 5909 | 41 | P18 | P22 | 59 | mainland | 0.43 | 4.59 | 9.67 | 0.42 |
| 5909_70_14 | 5909 | 70 | P18 | P22 | 59 | mainland | 0.43 | 4.61 | 11.93 | 0.64 |
| 6009_2_17 | 6009 | 2 | P5 | P3 | 60 | mainland | 0.39 | 4.64 | 7.99 | 0.41 |
| 6009_24_21 | 6009 | 24 | P5 | P3 | 60 | mainland | 0.33 | 4.61 | 8.20 | 0.40 |
| 6009_54_25 | 6009 | 54 | P5 | P3 | 60 | mainland | 0.46 | 4.50 | 10.56 | 0.51 |
| 6009_77_1 | 6009 | 77 | P5 | P3 | 60 | mainland | 0.46 | 4.57 | 9.18 | 0.45 |
| 6022_27_10 | 6022 | 27 | P5 | P3 | 60 | mainland | 0.47 | 4.54 | 11.54 | 0.59 |
| 6022_5_3 | 6022 | 5 | P5 | P3 | 60 | mainland | 0.31 | 4.74 | 5.12 | 0.25 |
| 6022_51_3 | 6022 | 51 | P5 | P3 | 60 | mainland | 0.51 | 4.58 | 9.54 | 0.49 |
| 6022_73_24 | 6022 | 73 | P5 | P3 | 60 | mainland | 0.64 | 4.37 | 18.51 | 0.69 |
| 6024_20_13 | 6024 | 20 | P5 | P3 | 60 | mainland | 0.48 | 4.61 | 9.55 | 0.53 |
| 6024_60_19 | 6024 | 60 | P5 | P3 | 60 | mainland | 0.41 | 4.69 | 8.30 | 0.40 |
| 6024_63_13 | 6024 | 63 | P5 | P3 | 60 | mainland | 0.33 | 4.67 | 8.49 | 0.43 |
| 6024_89_17 | 6024 | 89 | P5 | P3 | 60 | mainland | 0.50 | 4.37 | 11.75 | 0.55 |
| 6028_20_30 | 6028 | 20 | P5 | P3 | 60 | mainland | 0.49 | 4.37 | 11.21 | 0.47 |
| 6028_32_6 | 6028 | 32 | P5 | P3 | 60 | mainland | 0.44 | 4.54 | 8.59 | 0.37 |
| 6028_54_12 | 6028 | 54 | P5 | P3 | 60 | mainland | 0.39 | 5.10 | 6.02 | 0.30 |
| 6028_73_35 | 6028 | 73 | P5 | P3 | 60 | mainland | 0.48 | 4.65 | 9.53 | 0.48 |
| 605_38_29 | 605 | 38 | P7 | P28 | 6 | mixed | 0.44 | 4.57 | 8.80 | 0.44 |
| 605_42_4 | 605 | 42 | P7 | P28 | 6 | mixed | 0.37 | 4.48 | 11.49 | 0.58 |
| 605_63_12 | 605 | 63 | P7 | P28 | 6 | mixed | 0.36 | 4.68 | 8.77 | 0.45 |
| 605_7_13 | 605 | 7 | P7 | P28 | 6 | mixed | 0.55 | 4.38 | 17.32 | 0.61 |
| 606_16_7* | 606 | 16 | P7 | P28 | 6 | mixed | **0.44** | 4.63 | 9.08 | 0.43 |
| 606_37_30 | 606 | 37 | P7 | P28 | 6 | mixed | 0.51 | 4.36 | 12.39 | 0.54 |
| 606_44_30 | 606 | 44 | P7 | P28 | 6 | mixed | 0.48 | 4.43 | 11.36 | 0.57 |
| 606_73_8 | 606 | 73 | P7 | P28 | 6 | mixed | 0.46 | 4.78 | 8.47 | 0.41 |
| 607_23_22 | 607 | 23 | P7 | P28 | 6 | mixed | 0.39 | 4.69 | 8.85 | 0.40 |
| 607_46_24 | 607 | 46 | P7 | P28 | 6 | mixed | 0.37 | 4.51 | 10.88 | 0.50 |
| 607_5_32 | 607 | 5 | P7 | P28 | 6 | mixed | 0.39 | 4.62 | 9.50 | 0.44 |
| 607_77_17 | 607 | 77 | P7 | P28 | 6 | mixed | 0.51 | 4.42 | 12.08 | 0.53 |
| 611_13_7 | 611 | 13 | P7 | P28 | 6 | mixed | 0.35 | 4.82 | 8.30 | 0.40 |
| 611_40_9 | 611 | 40 | P7 | P28 | 6 | mixed | 0.55 | 4.48 | 15.26 | 0.68 |
| 611_54_14 | 611 | 54 | P7 | P28 | 6 | mixed | 0.46 | 4.81 | 9.59 | 0.48 |
| 611_67_21 | 611 | 67 | P7 | P28 | 6 | mixed | 0.47 | 4.45 | 11.11 | 0.52 |
| 6110_32_31 | 6110 | 32 | P19 | P3 | 61 | mainland | 0.37 | 4.59 | 8.12 | 0.35 |
| 6110_60_7 | 6110 | 60 | P19 | P3 | 61 | mainland | 0.45 | 4.50 | 11.23 | 0.52 |
| 6110_62_17 | 6110 | 62 | P19 | P3 | 61 | mainland | 0.47 | 4.42 | 11.89 | 0.51 |
| 6110_9_3 | 6110 | 9 | P19 | P3 | 61 | mainland | 0.56 | 4.38 | 15.01 | 0.61 |
| 6118_16_19 | 6118 | 16 | P19 | P3 | 61 | mainland | 0.40 | 4.83 | 6.75 | 0.28 |
| 6118_32_21 | 6118 | 32 | P19 | P3 | 61 | mainland | 0.44 | 4.41 | 11.30 | 0.48 |
| 6118_57_12 | 6118 | 57 | P19 | P3 | 61 | mainland | 0.46 | 4.56 | 9.15 | 0.45 |
| 6118_65_25 | 6118 | 65 | P19 | P3 | 61 | mainland | 0.41 | 4.72 | 8.85 | 0.45 |
| 6120_35_18 | 6120 | 35 | P19 | P3 | 61 | mainland | 0.52 | 4.37 | 14.60 | 0.63 |
| 6120_60_5 | 6120 | 60 | P19 | P3 | 61 | mainland | 0.37 | 4.71 | 8.57 | 0.40 |
| 6120_66_3 | 6120 | 66 | P19 | P3 | 61 | mainland | 0.42 | 4.54 | 10.05 | 0.45 |
| 6120_8_5 | 6120 | 8 | P19 | P3 | 61 | mainland | 0.51 | 4.51 | 11.57 | 0.54 |
| 6123_10_28 | 6123 | 10 | P19 | P3 | 61 | mainland | 0.58 | 4.55 | 15.34 | 0.67 |
| 6123_32_2 | 6123 | 32 | P19 | P3 | 61 | mainland | 0.39 | 4.68 | 7.78 | 0.39 |
| 6123_59_3 | 6123 | 59 | P19 | P3 | 61 | mainland | 0.36 | 4.79 | 9.06 | 0.38 |
| 6123_61_1 | 6123 | 61 | P19 | P3 | 61 | mainland | 0.44 | 4.58 | 9.02 | 0.45 |
| 6125_33_23 | 6125 | 33 | P19 | P3 | 61 | mainland | 0.37 | 5.04 | 5.76 | 0.31 |
| 6125_55_31 | 6125 | 55 | P19 | P3 | 61 | mainland | 0.46 | 4.71 | 9.90 | 0.55 |
| 6125_71_17 | 6125 | 71 | P19 | P3 | 61 | mainland | 0.55 | 4.30 | 13.56 | 0.60 |
| 6125_9_12 | 6125 | 9 | P19 | P3 | 61 | mainland | 0.46 | 4.61 | 10.44 | 0.43 |
| 616_18_3 | 616 | 18 | P7 | P28 | 6 | mixed | 0.42 | 4.99 | 6.81 | 0.35 |
| 616_21_13 | 616 | 21 | P7 | P28 | 6 | mixed | 0.38 | 4.71 | 7.62 | 0.35 |
| 616_55_29 | 616 | 55 | P7 | P28 | 6 | mixed | 0.50 | 4.59 | 9.89 | 0.48 |
| 616_76_12 | 616 | 76 | P7 | P28 | 6 | mixed | 0.41 | 4.62 | 9.83 | 0.51 |
| 6205_2_36 | 6205 | 2 | P5 | P30 | 62 | mainland | 0.40 | 4.74 | 7.78 | 0.39 |
| 6205_21_14 | 6205 | 21 | P5 | P30 | 62 | mainland | 0.49 | 4.50 | 13.59 | 0.61 |
| 6205_53_19 | 6205 | 53 | P5 | P30 | 62 | mainland | 0.49 | 4.64 | 10.35 | 0.46 |
| 6205_69_15 | 6205 | 69 | P5 | P30 | 62 | mainland | 0.54 | 4.31 | 12.84 | 0.62 |
| 6207_35_29 | 6207 | 35 | P5 | P30 | 62 | mainland | 0.53 | 4.36 | 11.81 | 0.52 |
| 6207_55_16 | 6207 | 55 | P5 | P30 | 62 | mainland | 0.40 | 4.90 | 7.87 | 0.43 |
| 6207_73_12 | 6207 | 73 | P5 | P30 | 62 | mainland | 0.55 | 4.54 | 11.10 | 0.51 |
| 6207_8_36 | 6207 | 8 | P5 | P30 | 62 | mainland | 0.43 | 4.91 | 8.15 | 0.34 |
| 6208_13_36 | 6208 | 13 | P5 | P30 | 62 | mainland | 0.44 | 4.56 | 9.49 | 0.44 |
| 6208_51_17 | 6208 | 51 | P5 | P30 | 62 | mainland | 0.46 | 4.68 | 8.24 | 0.44 |
| 6208_74_29 | 6208 | 74 | P5 | P30 | 62 | mainland | 0.48 | 4.70 | 9.81 | 0.52 |
| 6208_82_31 | 6208 | 82 | P5 | P30 | 62 | mainland | 0.41 | 4.67 | 7.83 | 0.37 |
| 6223_21_11* | 6223 | 21 | P5 | P30 | 62 | mainland | **0.44** | 4.88 | 7.07 | 0.36 |
| 6223_4_12 | 6223 | 4 | P5 | P30 | 62 | mainland | 0.40 | 4.40 | 10.02 | 0.37 |
| 6223_41_11 | 6223 | 41 | P5 | P30 | 62 | mainland | 0.39 | 4.57 | 8.91 | 0.42 |
| 6223_77_23 | 6223 | 77 | P5 | P30 | 62 | mainland | 0.45 | 4.44 | 10.94 | 0.53 |
| 716_17_26 | 716 | 17 | P18 | P16 | 7 | mainland | 0.40 | 4.74 | 7.78 | 0.37 |
| 716_31_2 | 716 | 31 | P18 | P16 | 7 | mainland | 0.41 | 4.52 | 10.82 | 0.49 |
| 716_43_19 | 716 | 43 | P18 | P16 | 7 | mainland | 0.39 | 4.59 | 9.35 | 0.45 |
| 716_67_24 | 716 | 67 | P18 | P16 | 7 | mainland | 0.43 | 4.56 | 10.28 | 0.49 |
| 717_33_13 | 717 | 33 | P18 | P16 | 7 | mainland | 0.36 | 5.05 | 6.25 | 0.30 |
| 717_48_33 | 717 | 48 | P18 | P16 | 7 | mainland | 0.47 | 4.65 | 9.77 | 0.41 |
| 717_6_24 | 717 | 6 | P18 | P16 | 7 | mainland | 0.38 | 4.52 | 8.94 | 0.39 |
| 717_67_11 | 717 | 67 | P18 | P16 | 7 | mainland | 0.50 | 4.39 | 13.56 | 0.54 |
| 721_12_19 | 721 | 12 | P18 | P16 | 7 | mainland | 0.44 | 4.70 | 8.33 | 0.35 |
| 721_36_9 | 721 | 36 | P18 | P16 | 7 | mainland | 0.49 | 4.56 | 9.02 | 0.43 |
| 721_47_26 | 721 | 47 | P18 | P16 | 7 | mainland | 0.48 | 4.54 | 10.88 | 0.47 |
| 721_70_36 | 721 | 70 | P18 | P16 | 7 | mainland | 0.40 | 4.58 | 9.41 | 0.43 |
| 723_18_16 | 723 | 18 | P18 | P16 | 7 | mainland | 0.45 | 4.84 | 10.08 | 0.52 |
| 723_55_20 | 723 | 55 | P18 | P16 | 7 | mainland | 0.48 | 4.49 | 11.33 | 0.52 |
| 723_78_31 | 723 | 78 | P18 | P16 | 7 | mainland | 0.51 | 4.42 | 11.15 | 0.58 |
| 723_94_33 | 723 | 94 | P18 | P16 | 7 | mainland | 0.57 | 4.66 | 9.49 | 0.45 |
| 724_100_31 | 724 | 100 | P18 | P16 | 7 | mainland | 0.43 | 4.46 | 10.13 | 0.49 |
| 724_47_32 | 724 | 47 | P18 | P16 | 7 | mainland | 0.49 | 4.24 | 10.23 | 0.46 |
| 724_6_21 | 724 | 6 | P18 | P16 | 7 | mainland | 0.36 | 4.79 | 8.84 | 0.45 |
| 724_72_13* | 724 | 72 | P18 | P16 | 7 | mainland | **0.44** | 4.58 | 13.17 | 0.50 |
| 725_12_22 | 725 | 12 | P18 | P16 | 7 | mainland | 0.47 | 4.55 | 9.77 | 0.50 |
| 725_31_32 | 725 | 31 | P18 | P16 | 7 | mainland | 0.45 | 4.61 | 9.65 | 0.48 |
| 725_54_34 | 725 | 54 | P18 | P16 | 7 | mainland | 0.54 | 4.81 | 7.94 | 0.35 |
| 725_62_8 | 725 | 62 | P18 | P16 | 7 | mainland | 0.59 | 4.45 | 11.84 | 0.47 |
| 804_12_10 | 804 | 12 | P2 | P4 | 8 | mixed | 0.45 | 4.68 | 8.75 | 0.42 |
| 804_25_2 | 804 | 25 | P2 | P4 | 8 | mixed | 0.44 | 4.84 | 9.01 | 0.41 |
| 804_51_24 | 804 | 51 | P2 | P4 | 8 | mixed | 0.51 | 4.40 | 12.80 | 0.59 |
| 804_73_10* | 804 | 73 | P2 | P4 | 8 | mixed | **0.44** | 4.69 | 10.39 | 0.42 |
| 812_2_35 | 812 | 2 | P2 | P4 | 8 | mixed | 0.46 | 4.66 | 8.05 | 0.35 |
| 812_30_34 | 812 | 30 | P2 | P4 | 8 | mixed | 0.46 | 4.57 | 10.13 | 0.49 |
| 812_51_36 | 812 | 51 | P2 | P4 | 8 | mixed | 0.39 | 4.71 | 8.21 | 0.37 |
| 812_76_7 | 812 | 76 | P2 | P4 | 8 | mixed | 0.41 | 4.90 | 8.24 | 0.38 |
| 816_21_17 | 816 | 21 | P2 | P4 | 8 | mixed | 0.47 | 4.50 | 10.43 | 0.39 |
| 816_47_5 | 816 | 47 | P2 | P4 | 8 | mixed | 0.45 | 4.54 | 8.75 | 0.39 |
| 816_65_12 | 816 | 65 | P2 | P4 | 8 | mixed | 0.41 | 4.63 | 8.90 | 0.41 |
| 816_7_3* | 816 | 7 | P2 | P4 | 8 | mixed | **0.44** | 4.48 | 14.75 | 0.69 |
| 818_28_26 | 818 | 28 | P2 | P4 | 8 | mixed | 0.50 | 4.48 | 9.69 | 0.48 |
| 818_51_6 | 818 | 51 | P2 | P4 | 8 | mixed | 0.43 | 4.66 | 7.39 | 0.31 |
| 818_7_16* | 818 | 7 | P2 | P4 | 8 | mixed | **0.44** | 4.62 | 12.06 | 0.50 |
| 818_72_33 | 818 | 72 | P2 | P4 | 8 | mixed | 0.53 | 4.35 | 12.75 | 0.50 |
| 822_16_23 | 822 | 16 | P2 | P4 | 8 | mixed | 0.39 | 4.92 | 7.23 | 0.37 |
| 822_35_28 | 822 | 35 | P2 | P4 | 8 | mixed | 0.47 | 4.75 | 9.02 | 0.44 |
| 822_58_20 | 822 | 58 | P2 | P4 | 8 | mixed | 0.41 | 4.69 | 8.52 | 0.42 |
| 822_65_36 | 822 | 65 | P2 | P4 | 8 | mixed | 0.39 | 4.56 | 10.66 | 0.51 |

*^†^Full pedigrees were used however these are the confidential commercial property of the Radiata Pine Breeding Company*

**Table S2** Comparison of soil variables predicted using diffuse reflectance mid-infrared spectroscopy (DRIFTS) with traditional laboratory analyses (n = 53). Goodness of fit statistics include residual mean sum of squares (rmse), bias, R^2^, and Lin’s **concordance correlation coefficient (ccc).**

| **Variable** | **rmse** | **bias** | **R**^2^ | **ccc** |
| --- | --- | --- | --- | --- |
| total C | 0.94 | -0.54 | 0.9 | 0.93 |
| total N | 0.13 | 0.12 | 0.87 | 0.55 |
| pH | 0.12 | -0.02 | 0.82 | 0.86 |

**Table S3** Mean relative abundance (RA) (not spatially corrected) and heritability of the Pinus radiata root core bacterial microbiome. Bacterial ASVs present in ≥ 80% of root samples (n = 505) sampled from clonal replicates of 132 unique P. radiata genotypes across 28 full-sib families, showing broad-sense (H^2^) and narrow-sense heritability (h^2^).

| **ASV** | **Phylum** | **Class** | **Order** | **Family** | **Genus** | **Mean RA (%)** | **H^2^** | **SE (H^2^)** | **Z(H^2^)** | **h^2^** | **SE (h^2^)** | **Z(h^2^)** |
| --- | --- | --- | --- | --- | --- | --- | --- | --- | --- | --- | --- | --- |
| ASV_2 | *Proteobacteria* | *Alphaproteobacteria* | *Rhizobiales* | *Bradyrhizobiaceae* | *Bradyrhizobium* | 4.34 | 0.01 | 0.04 | 0.29 | 0.00 | 0.07 | 0.00 |
| ASV_3 | *Proteobacteria* | *Alphaproteobacteria* | *Rhizobiales* | *Bradyrhizobiaceae* | *Bradyrhizobium* | 2.85 | 0.04 | 0.04 | 0.88 | 0.01 | 0.03 | 0.31 |
| ASV_6 | *Proteobacteria* | *Betaproteobacteria* | *Burkholderiales* | *Burkholderiaceae* | *Paraburkholderia* | 2.39 | 0.05 | 0.04 | 1.29 | 0.05 | 0.04 | 1.29 |
| ASV_7 | *Firmicutes* | *Bacilli* | *Bacillales* | *Bacillaceae_1* | NA | 0.62 | 0.04 | 0.04 | 1.15 | 0.04 | 0.04 | 1.15 |
| ASV_8 | *Proteobacteria* | *Gammaproteobacteria* | *Nevskiales* | *Steroidobacteraceae* | *Povalibacter* | 1.10 | 0.00 | 0.04 | 0.00 | 0.00 | 0.00 | **26.16** |
| ASV_9 | *Proteobacteria* | *Alphaproteobacteria* | *Rhizobiales* | *Bradyrhizobiaceae* | NA | 1.13 | 0.03 | 0.04 | 0.81 | 0.02 | 0.06 | 0.38 |
| ASV_10 | *Proteobacteria* | *Alphaproteobacteria* | *Rhizobiales* | *Roseiarcaceae* | *Roseiarcus* | 0.66 | 0.07 | 0.04 | **1.76** | 0.07 | 0.04 | **1.76** |
| ASV_11 | *Actinobacteria* | *Actinobacteria* | *Streptosporangiales* | *Thermomonosporaceae* | *Actinoallomurus* | 0.74 | 0.06 | 0.04 | 1.45 | 0.05 | 0.06 | 0.96 |
| ASV_12 | *Acidobacteria* | *Acidobacteria_Gp2* | *Gp2* | NA | NA | 1.14 | 0.00 | 0.03 | 0.00 | 0.00 | 0.03 | 0.00 |
| ASV_13 | *Proteobacteria* | *Gammaproteobacteria* | *Gammaproteobacteria_incertae_sedis* | *Acidibacter* | NA | 1.38 | 0.02 | 0.03 | 0.57 | 0.00 | 0.04 | 0.00 |
| ASV_15 | *Actinobacteria* | *Actinobacteria* | *Streptomycetales* | *Streptomycetaceae* | *Streptacidiphilus* | 1.51 | 0.01 | 0.04 | 0.15 | 0.00 | 0.00 | **25.97** |
| ASV_16 | *Proteobacteria* | *Gammaproteobacteria* | *Gammaproteobacteria_incertae_sedis* | *Acidibacter* | NA | 1.43 | 0.08 | 0.04 | **1.89** | 0.00 | 0.00 | **21.03** |
| ASV_19 | *Proteobacteria* | *Gammaproteobacteria* | *Nevskiales* | *Steroidobacteraceae* | *Povalibacter* | 0.60 | 0.05 | 0.04 | 1.20 | 0.03 | 0.08 | 0.40 |
| ASV_22 | *Actinobacteria* | *Thermoleophilia* | *Solirubrobacterales* | *Conexibacteraceae* | *Conexibacter* | 0.45 | 0.04 | 0.04 | 0.87 | 0.00 | 0.00 | **24.03** |
| ASV_23 | *Proteobacteria* | *Betaproteobacteria* | *Burkholderiales* | *Burkholderiaceae* | *Caballeronia* | 1.00 | 0.02 | 0.03 | 0.78 | 0.00 | 0.05 | 0.00 |
| ASV_24 | *Proteobacteria* | *Gammaproteobacteria* | *Gammaproteobacteria_incertae_sedis* | *Acidibacter* | NA | 1.04 | 0.03 | 0.04 | 0.64 | 0.00 | 0.06 | 0.00 |
| ASV_25 | *Actinobacteria* | *Actinobacteria* | *Mycobacteriales* | *Mycobacteriaceae* | *Mycobacterium* | 0.55 | 0.05 | 0.04 | 1.23 | 0.00 | 0.00 | **23.04** |
| ASV_26 | *Planctomycetes* | *Planctomycetacia* | *Planctomycetales* | *Isosphaeraceae* | NA | 0.44 | 0.05 | 0.04 | 1.19 | 0.03 | 0.05 | 0.75 |
| ASV_28 | *Proteobacteria* | *Alphaproteobacteria* | *Rhizobiales* | NA | NA | 0.54 | 0.01 | 0.04 | 0.36 | 0.00 | 0.05 | 0.09 |
| ASV_29 | *Proteobacteria* | *Alphaproteobacteria* | *Rhizobiales* | *Bradyrhizobiaceae* | *Bradyrhizobium* | 0.65 | 0.00 | 0.03 | 0.00 | 0.00 | 0.03 | 0.00 |
| ASV_30 | *Proteobacteria* | *Deltaproteobacteria* | *Myxococcales* | NA | NA | 0.72 | 0.00 | 0.02 | 0.23 | 0.00 | 0.00 | **55.81** |
| ASV_33 | *Proteobacteria* | *Gammaproteobacteria* | *Gammaproteobacteria_incertae_sedis* | *Acidibacter* | NA | 0.21 | 0.01 | 0.02 | 0.43 | 0.01 | 0.02 | 0.43 |
| ASV_37 | *Actinobacteria* | *Actinobacteria* | *Streptosporangiales* | *Thermomonosporaceae* | NA | 0.73 | 0.00 | 0.02 | 0.00 | 0.00 | 0.00 | **55.47** |
| ASV_38 | *Actinobacteria* | *Actinobacteria* | *Catenulisporales* | *Actinospicaceae* | *Actinospica* | 0.68 | 0.06 | 0.03 | **1.89** | 0.00 | 0.00 | **28.43** |
| ASV_39 | *Acidobacteria* | *Acidobacteria_Gp1* | *Gp1* | NA | NA | 0.31 | 0.02 | 0.02 | 0.92 | 0.00 | 0.00 | **42.47** |
| ASV_44 | *Proteobacteria* | *Alphaproteobacteria* | *Rhizobiales* | *Bradyrhizobiaceae* | NA | 0.26 | **0.11** | 0.05 | **2.17** | **0.11** | 0.08 | 1.41 |
| ASV_46 | *Firmicutes* | *Bacilli* | *Bacillales* | *Bacillaceae_1* | *Bacillus* | 0.20 | 0.02 | 0.03 | 0.60 | 0.02 | 0.03 | 0.60 |
| ASV_47 | *Proteobacteria* | *Alphaproteobacteria* | *Micropepsales* | *Micropepsaceae* | *Micropepsis* | 0.49 | 0.02 | 0.02 | 0.91 | 0.00 | 0.00 | **45.32** |
| ASV_50 | *Acidobacteria* | *Acidobacteria_Gp2* | *Gp2* | NA | NA | 0.41 | 0.00 | 0.04 | 0.06 | 0.00 | 0.00 | **26.32** |
| ASV_53 | *Planctomycetes* | *Planctomycetacia* | *Planctomycetales* | *Gemmataceae* | NA | 0.17 | 0.02 | 0.04 | 0.56 | 0.00 | 0.00 | **24.65** |
| ASV_59 | *Acidobacteria* | *Acidobacteria_Gp1* | *Gp1* | NA | NA | 0.14 | 0.06 | 0.04 | 1.41 | 0.05 | 0.05 | 1.00 |
| ASV_64 | *Proteobacteria* | *Alphaproteobacteria* | *Rhizobiales* | *Bradyrhizobiaceae* | NA | 0.42 | 0.00 | 0.02 | 0.19 | 0.00 | 0.04 | 0.00 |
| ASV_70 | *Proteobacteria* | *Alphaproteobacteria* | *Rhizobiales* | *Bradyrhizobiaceae* | *Bradyrhizobium* | 0.39 | 0.00 | 0.02 | 0.00 | 0.00 | 0.02 | 0.00 |
| ASV_72 | *Bacteroidetes* | *Chitinophagia* | *Chitinophagales* | *Chitinophagaceae* | *Flavitalea* | 0.53 | 0.01 | 0.03 | 0.49 | 0.01 | 0.03 | 0.49 |
| ASV_79 | *Chloroflexi* | *Ktedonobacteria* | *Ktedonobacterales* | *Ktedonobacteraceae* | *Ktedonobacter* | 0.48 | 0.08 | 0.04 | **1.76** | 0.01 | 0.06 | 0.23 |
| ASV_81 | *Proteobacteria* | *Alphaproteobacteria* | *Rhodospirillales* | *Reyranellaceae* | *Reyranella* | 0.24 | 0.00 | NA | NA | 0.00 | 0.00 | **1.24 x 10^9^** |
| ASV_85 | *Proteobacteria* | *Alphaproteobacteria* | *Rhizobiales* | *Bradyrhizobiaceae* | NA | 0.47 | 0.00 | 0.02 | 0.00 | 0.00 | 0.00 | **54.90** |
| ASV_96 | *Actinobacteria* | *Actinobacteria* | *Streptosporangiales* | NA | NA | 0.40 | 0.01 | 0.04 | 0.33 | 0.00 | 0.00 | **25.58** |
| ASV_97 | *Proteobacteria* | *Alphaproteobacteria* | *Rhodospirillales* | *Azospirillaceae* | NA | 0.18 | 0.00 | 0.02 | 0.00 | 0.00 | 0.02 | 0.00 |
| ASV_102 | *Acidobacteria* | *Acidobacteria_Gp1* | *Acidobacterium* | NA | NA | 0.35 | 0.00 | 0.04 | 0.00 | 0.00 | 0.00 | **26.50** |
| ASV_103 | *Acidobacteria* | *Acidobacteria_Gp3* | *Candidatus_Solibacter* | NA | NA | 0.18 | 0.03 | 0.04 | 0.85 | 0.00 | 0.00 | **23.69** |
| ASV_104 | *Planctomycetes* | *Planctomycetacia* | *Planctomycetales* | *Isosphaeraceae* | *Tundrisphaera* | 0.36 | 0.01 | 0.03 | 0.50 | 0.01 | 0.04 | 0.21 |
| ASV_108 | *Proteobacteria* | *Betaproteobacteria* | *Burkholderiales* | *Comamonadaceae* | NA | 0.37 | 0.03 | 0.04 | 0.79 | 0.01 | 0.05 | 0.30 |
| ASV_112 | *Proteobacteria* | *Deltaproteobacteria* | *Myxococcales* | *Polyangiaceae* | NA | 0.26 | 0.00 | 0.02 | 0.25 | 0.00 | 0.00 | **53.45** |
| ASV_115 | *Chloroflexi* | *Ktedonobacteria* | *Ktedonobacterales* | NA | NA | 0.38 | 0.00 | 0.04 | 0.05 | 0.00 | 0.03 | 0.00 |
| ASV_123 | *Acidobacteria* | *Acidobacteria_Gp3* | *Gp3* | NA | NA | 0.15 | 0.01 | 0.04 | 0.36 | 0.00 | 0.05 | 0.00 |
| ASV_131 | *Proteobacteria* | *Alphaproteobacteria* | *Rhizobiales* | *Xanthobacteraceae* | *Labrys* | 0.20 | 0.00 | 0.02 | 0.00 | 0.00 | 0.00 | **55.40** |
| ASV_156 | *Proteobacteria* | *Alphaproteobacteria* | *Caulobacterales* | *Caulobacteraceae* | *Phenylobacterium* | 0.15 | 0.00 | 0.04 | 0.11 | 0.00 | 0.00 | **26.03** |
| ASV_163 | *Actinobacteria* | *Actinobacteria* | *Mycobacteriales* | *Mycobacteriaceae* | *Mycobacterium* | 0.19 | 0.00 | 0.04 | 0.00 | 0.00 | 0.00 | **26.13** |
| ASV_183 | *Proteobacteria* | *Deltaproteobacteria* | *Myxococcales* | *Polyangiaceae* | NA | 0.22 | 0.00 | 0.02 | 0.22 | 0.00 | 0.00 | **56.75** |
| ASV_209 | *Proteobacteria* | *Deltaproteobacteria* | *Myxococcales* | *Polyangiaceae* | NA | 0.16 | 0.00 | 0.03 | 0.19 | 0.00 | 0.03 | 0.19 |
| ASV_210 | *Acidobacteria* | *Acidobacteria_Gp3* | *Gp3* | NA | NA | 0.18 | 0.01 | 0.04 | 0.37 | 0.00 | 0.00 | **24.70** |
| ASV_218 | *Proteobacteria* | *Alphaproteobacteria* | *Rhodospirillales* | *Acetobacteraceae* | *Acidisoma* | 0.14 | 0.00 | 0.04 | 0.06 | 0.00 | 0.00 | **25.82** |

*SE: standard error; Z: z-score (> |1.65| in bold)*

**Table S4** Mean relative abundance (RA) (not spatially corrected) and heritability of the Pinus radiata root core fungal microbiome. Fungal ASVs present in ≥ 80% of root samples (n = 500) sampled from clonal replicates of 132 unique P. radiata genotypes across 28 full-sib families, showing broad-sense (H^2^) and narrow-sense heritability (h^2^).

| **ASV** | **Phylum** | **Class** | **Order** | **Family** | **Genus** | **Species** | **Mean RA (%)** | **H^2^** | **SE (H^2^)** | **Z(H^2^)** | **h^2^** | **SE (h^2^)** | **Z(h^2^)** |
| --- | --- | --- | --- | --- | --- | --- | --- | --- | --- | --- | --- | --- | --- |
| ASV_1 | *Basidiomycota* | *Tremellomycetes* | *Tremellales* | *Trimorphomycetaceae* | *Saitozyma* | *podzolica* | 0.56 | 0.06 | 0.03 | **1.80** | 0.00 | 0.00 | **28.28** |
| ASV_2 | *Ascomycota* | *Leotiomycetes* | *Helotiales* | *Vibrisseaceae* | *Phialocephala* | *fortinii* | 7.84 | 0.07 | 0.04 | **1.66** | 0.00 | 0.06 | 0.00 |
| ASV_4 | *Ascomycota* | NA | NA | NA | NA | NA | 5.55 | 0.00 | 0.04 | 0.03 | 0.00 | 0.00 | **26.10** |
| ASV_5 | *Mortierellomycota* | *Mortierellomycetes* | *Mortierellales* | *Mortierellaceae* | *Mortierella* | *humilis* | 0.48 | 0.06 | 0.04 | 1.55 | 0.06 | 0.04 | 1.55 |
| ASV_13 | *Ascomycota* | *Archaeorhizomycetes* | *Archaeorhizomycetales* | *Archaeorhizomycetaceae* | *Archaeorhizomyces* | *borealis* | 2.28 | 0.04 | 0.04 | 1.06 | 0.02 | 0.04 | 0.53 |
| ASV_15 | *Ascomycota* | *Archaeorhizomycetes* | *Archaeorhizomycetales* | *Archaeorhizomycetaceae* | *Archaeorhizomyces* | *borealis* | 2.27 | 0.08 | 0.04 | **1.87** | 0.08 | 0.04 | **1.87** |
| ASV_23 | *Basidiomycota* | *Tremellomycetes* | *Filobasidiales* | *Piskurozymaceae* | *Solicoccozyma* | *terricola* | 0.11 | 0.03 | 0.04 | 0.67 | 0.03 | 0.06 | 0.48 |
| ASV_25 | *Ascomycota* | NA | NA | NA | NA | NA | 1.77 | 0.05 | 0.04 | 1.17 | 0.00 | 0.10 | 0.00 |
| ASV_28 | *Basidiomycota* | *Microbotryomycetes* | *Microbotryomycetes_ord_*  *Incertae_sedis* | *Chrysozymaceae* | *Slooffia* | *cresolica* | 0.74 | 0.07 | 0.04 | **1.67** | 0.05 | 0.06 | 0.90 |
| ASV_30 | *Ascomycota* | *Dothideomycetes* | *Dothideomycetes_ord_*  *Incertae_sedis* | *Dothideomycetes_fam_*  *Incertae_sedis* | *Septonema* | *fasciculare* | 1.94 | 0.01 | 0.03 | 0.47 | 0.01 | 0.03 | 0.47 |
| ASV_33 | *Ascomycota* | *Eurotiomycetes* | *Chaetothyriales* | *Herpotrichiellaceae* | NA | NA | 1.56 | 0.03 | 0.03 | 1.06 | 0.00 | 0.05 | 0.00 |
| ASV_74 | *Ascomycota* | *Sordariomycetes* | *Hypocreales* | *Nectriaceae* | *Ilyonectria* | NA | 0.14 | 0.07 | 0.05 | 1.50 | 0.00 | 0.11 | 0.00 |
|  |  |  |  |  |  |  |  |  |  |  |  |  |  |

*SE: standard error; Z: z-score (> |1.65| in bold)*

**Table S5** Results from testing for homogeneity of multivariate dispersion of bacterial and fungal root microbiomes across 132 unique genotypes of Pinus radiata, across 3 ancestry groups (mainland, island, and mixed) and 28 full-sib families.

|  | **Bacteria *p*_perm_^a^** | **Fungi *p*_perm_^a^** |
| --- | --- | --- |
| No. samples | 505 | 500 |
| Ancestry | 0.198 | 0.054 |
| Family | 0.832 | 0.689 |
| Genotype | 0.928 | 0.329 |
| Sequencing batch | 0.859 | 0.874 |
|  |  |  |

*^a^ p_perm_ is the probability statistic derived from 999 permutations of residuals under a reduced model*

**Table S6** PERMANOVA results for testing influence of ancestry on bacterial and fungal community composition in Pinus radiata roots. Host family (random effect) was nested within ancestry (fixed effect), and host genotype (random effect) was nested in family. Covariates of sequencing batch and soil PC1 were fitted first on spatially-corrected ASV counts for bacterial and fungal root microbiomes across 22 full-sib families (six families of multiple ancestry types were removed) of 102 P. radiata genotypes. **Families with progeny of more than one ancestry type were removed.** Significant values (p_perm_ < 0.05) are bolded. Type I (sequential) sum of squares.

|  | **Bacteria** | | **Fungi** | |  |
| --- | --- | --- | --- | --- | --- |
|  | **n = 389** | | **n = 388** | |  |
|  | **√CV^a^** | ***p*_perm_^b^** | **√CV^a^** | ***p*_perm_^b^** | |
| Sequencing batch | 1.94 | 0.001 | 2.47 | **0.001** | |
| soil PC1^c^ | 3.92 | 0.001 | 2.61 | **0.001** | |
| Ancestry | 0.94 | 0.239 | -0.61 | 0.604 | |
| Family (within Ancestry) | 0.82 | 0.302 | 1.57 | **0.074** | |
| Residual | 21.14 |  | 21.17 |  | |

*^a^√CV is the square root of the component of variation associated with each term*

*^b^ p_perm_ is the probability statistic derived from 999 permutations of residuals under a reduced model*

*^c^ First principal component coordinates from PCA of soil pH, gravimetric moisture, total carbon and total nitrogen*

**Table S7** Bacterial ASVs with significant broad-sense heritability (H^2^) > 0.2 across 132 different genotypes of Pinus radiata from 28 full-sib families (n = 505).

| **ASV** | **Phylum** | **Class** | **Order** | **Family** | **Genus** | **H^2^** | **SE(H^2^)** | **Z(H^2^)** | **h^2^** | **SE(h^2^)** | **Z(H^2^)** |
| --- | --- | --- | --- | --- | --- | --- | --- | --- | --- | --- | --- |
| ASV_255 | *Proteobacteria* | *Betaproteobacteria* | *Burkholderiales* | *Burkholderiaceae* | *Ralstonia* | 0.23 | 0.05 | **4.65** | 0.00 | 0.05 | 0.08 |
| ASV_511 | *Actinobacteria* | *Actinobacteria* | *Micrococcales* | *Micrococcaceae* | *Micrococcus* | **0.21** | 0.06 | **3.80** | 0.00 | 0.15 | 0.00 |
| ASV_583 | *Bacteroidetes* | *Flavobacteriia* | *Flavobacteriales* | *Weeksellaceae* | *Cloacibacterium* | **0.40** | 0.05 | **7.87** | 0.00 | 0.00 | **11.98** |
| ASV_2839 | *Verrucomicrobia* | *Spartobacteria* | NA | NA | NA | 0.46 | 0.05 | **9.43** | 0.00 | 0.00 | **10.86** |
| ASV_3469 | *Actinobacteria* | *Actinobacteria* | *Streptomycetales* | *Streptomycetaceae* | NA | 0.27 | 0.05 | **5.47** | 0.03 | 0.07 | 0.45 |
| ASV_3715 | *Proteobacteria* | *Deltaproteobacteria* | *Myxococcales* | *Polyangiaceae* | *Aetherobacter* | 0.21 | 0.05 | **4.21** | 0.02 | 0.05 | 0.46 |
| ASV_4146 | *Verrucomicrobia* | *Subdivision3* | *NA* | *NA* | NA | 0.29 | 0.05 | **5.67** | 0.00 | 0.00 | **13.59** |
| ASV_4395^*^ | *Bacteroidetes* | *Chitinophagia* | *Chitinophagales* | *Chitinophagaceae* | NA | 0.25 | 0.06 | **4.06** | **0.25** | **0.06** | **4.06** |
| ASV_4750 | *Bacteroidetes* | NA | NA | NA | NA | 0.26 | 0.05 | **5.35** | 0.01 | 0.06 | 0.23 |
| ASV_4869 | *Firmicutes* | *Bacilli* | *Bacillales* | *Paenibacillaceae_1* | *Paenibacillus* | 0.20 | 0.05 | **4.12** | 0.02 | 0.08 | 0.26 |
| ASV_4972 | *Proteobacteria* | *Alphaproteobacteria* | *Rhodospirillales* | NA | NA | 0.36 | 0.05 | **6.90** | 0.03 | 0.07 | 0.37 |
| ASV_5356 | *Acidobacteria* | *Acidobacteria_Gp1* | *Gp1* | NA | NA | 0.30 | 0.05 | **6.00** | 0.03 | 0.08 | 0.37 |
| ASV_5441 | *Planctomycetes* | *Planctomycetacia* | *Planctomycetales* | *Isosphaeraceae* | *Aquisphaera* | 0.21 | 0.05 | **4.31** | 0.00 | 0.00 | **16.24** |
| ASV_5528 | *Proteobacteria* | *Alphaproteobacteria* | *Micropepsales* | *Micropepsaceae* | *Rhizomicrobium* | 0.33 | 0.06 | **5.49** | 0.03 | 0.07 | 0.35 |
| ASV_5534 | *Chlamydiae* | *Chlamydiia* | *Chlamydiales* | *Simkaniaceae* | *Simkania* | 0.22 | 0.05 | **4.60** | 0.00 | 0.00 | **16.41** |
| ASV_5628^*^ | *Proteobacteria* | *Alphaproteobacteria* | *Caulobacterales* | *Caulobacteraceae* | NA | 0.24 | 0.06 | **3.92** | **0.24** | **0.06** | **3.92** |
| ASV_5935 | *Planctomycetes* | *Planctomycetacia* | *Planctomycetales* | *Isosphaeraceae* | *Tundrisphaera* | 0.31 | 0.06 | **5.40** | 0.00 | 0.00 | **11.85** |
| ASV_7029 | *Proteobacteria* | *Alphaproteobacteria* | *Micropepsales* | *Micropepsaceae* | *Micropepsis* | 0.29 | 0.05 | **5.62** | 0.07 | 0.12 | 0.59 |
| ASV_7030 | *Chloroflexi* | *Ktedonobacteria* | *Ktedonobacterales* | *Ktedonobacteraceae* | *Ktedonobacter* | 0.25 | 0.05 | **4.62** | 0.03 | 0.06 | 0.41 |
| ASV_7288 | *Proteobacteria* | *Betaproteobacteria* | *Burkholderiales* | *Burkholderiaceae* | *Paraburkholderia* | 0.29 | 0.05 | **5.92** | 0.00 | 0.05 | 0.00 |
| ASV_7624 | *Chlamydiae* | *Chlamydiia* | *Chlamydiales* | *Parachlamydiaceae* | NA | 0.32 | 0.05 | **6.52** | 0.00 | 0.06 | 0.00 |
| ASV_8380 | *Bacteroidetes* | *Chitinophagia* | *Chitinophagales* | *Chitinophagaceae* | NA | 0.23 | 0.05 | **4.65** | 0.03 | 0.09 | 0.40 |
| ASV_8828 | *Proteobacteria* | *Alphaproteobacteria* | NA | NA | NA | 0.33 | 0.05 | **6.63** | 0.00 | 0.00 | **13.63** |
| ASV_8977 | *Proteobacteria* | *Gammaproteobacteria* | *Xanthomonadales* | *Rhodanobacteraceae* | *Rudaea* | 0.23 | 0.05 | **4.85** | 0.02 | 0.05 | 0.40 |
| ASV_9032 | *Chloroflexi* | *Ktedonobacteria* | *Ktedonobacterales* | NA | NA | 0.20 | 0.05 | **4.25** | 0.01 | 0.07 | 0.10 |
| ASV_9459 | *Acidobacteria* | *Acidobacteria_Gp3* | *Gp3* | NA | NA | 0.33 | 0.06 | **5.38** | 0.03 | 0.08 | 0.36 |
| ASV_9982 | *Proteobacteria* | *Alphaproteobacteria* | *Rhodospirillales* | *Acetobacteraceae* | *Acidisoma* | 0.26 | 0.05 | **5.05** | 0.05 | 0.11 | 0.45 |
| ASV_10247 | *Acidobacteria* | *Acidobacteria_Gp1* | *Gp1* | NA | NA | 0.21 | 0.05 | **4.45** | 0.00 | 0.00 | **16.53** |
| ASV_10536 | *Proteobacteria* | NA | NA | NA | NA | 0.23 | 0.05 | **4.51** | 0.00 | 0.00 | **14.93** |
| ASV_10699 | *Chlamydiae* | *Chlamydiia* | *Chlamydiales* | *Parachlamydiaceae* | *Neochlamydia* | 0.24 | 0.05 | **5.01** | 0.00 | 0.05 | 0.00 |
| ASV_11301 | *Gemmatimonadetes* | *Gemmatimonadetes* | *Gemmatimonadales* | *Gemmatimonadaceae* | *NA* | 0.30 | 0.05 | **6.15** | 0.00 | 0.05 | 0.00 |
| ASV_12984 | *Planctomycetes* | *Planctomycetacia* | *Pirellulales* | *Lacipirellulaceae* | *Bythopirellula* | 0.23 | 0.05 | **4.79** | 0.00 | 0.00 | **15.94** |
| ASV_13830 | *Bacteroidetes* | *Chitinophagia* | *Chitinophagales* | *Chitinophagaceae* | *Paraflavitalea* | 0.33 | 0.06 | **5.49** | 0.03 | 0.07 | 0.35 |
| ASV_13989 | *Proteobacteria* | *Gammaproteobacteria* | NA | NA | NA | 0.21 | 0.05 | **4.44** | 0.01 | 0.06 | 0.23 |
| ASV_14855 | *Planctomycetes* | *Planctomycetacia* | *Planctomycetales* | *Gemmataceae* | *Zavarzinella* | 0.32 | 0.06 | **5.37** | 0.00 | 0.00 | **11.31** |
| ASV_16339 | *Proteobacteria* | NA | NA | NA | NA | 0.34 | 0.06 | **5.59** | 0.03 | 0.08 | 0.34 |
| ASV_16675 | *Proteobacteria* | *Gammaproteobacteria* | *Xanthomonadales* | *Rhodanobacteraceae* | *Dokdonella* | 0.33 | 0.06 | **5.49** | 0.03 | 0.07 | 0.35 |
| ASV_16794 | *Planctomycetes* | *Planctomycetacia* | *Planctomycetales* | NA | NA | 0.32 | 0.06 | **5.37** | 0.00 | 0.00 | **11.31** |
| ASV_17016 | *Planctomycetes* | *Planctomycetacia* | *Planctomycetales* | *Gemmataceae* | NA | 0.34 | 0.06 | **5.59** | 0.03 | 0.08 | 0.34 |
| ASV_17408 | *Chlamydiae* | *Chlamydiia* | *Chlamydiales* | *Simkaniaceae* | *Simkania* | 0.33 | 0.06 | **5.38** | 0.03 | 0.08 | 0.36 |
| ASV_19179 | *Planctomycetes* | *Planctomycetacia* | *Planctomycetales* | *Isosphaeraceae* | NA | 0.33 | 0.06 | **5.38** | 0.03 | 0.08 | 0.36 |
| ASV_19181 | *Proteobacteria* | *Alphaproteobacteria* | *Rhodospirillales* | *Rhodospirillaceae* | *Ferruginivarius* | 0.33 | 0.06 | **5.49** | 0.03 | 0.07 | 0.35 |
| ASV_20206 | *Proteobacteria* | NA | NA | NA | NA | 0.31 | 0.05 | **6.25** | 0.01 | 0.06 | 0.12 |
| ASV_20386 | *Firmicutes* | *Clostridia* | *Clostridiales* | *Ruminococcaceae* | *Clostridium_IV* | 0.34 | 0.06 | **5.59** | 0.03 | 0.08 | 0.34 |
| ASV_21701 | *Acidobacteria* | *Acidobacteria_Gp16* | *Gp16* | NA | NA | 0.32 | 0.06 | **5.37** | 0.00 | 0.00 | **11.31** |
| ASV_22619 | *Parcubacteria* | NA | NA | NA | NA | 0.33 | 0.06 | **5.38** | 0.03 | 0.08 | 0.36 |
| ASV_22620 | *candidate_division_*  *WPS-1* | NA | NA | NA | NA | 0.33 | 0.06 | **5.49** | 0.03 | 0.07 | 0.35 |
| ASV_23501 | *Proteobacteria* | *Gammaproteobacteria* | NA | NA | NA | 0.33 | 0.06 | **5.38** | 0.03 | 0.08 | 0.36 |
| ASV_23502 | *Chlamydiae* | *Chlamydiia* | *Chlamydiales* | *Simkaniaceae* | *Simkania* | 0.33 | 0.06 | **5.38** | 0.03 | 0.08 | 0.36 |
| ASV_23503 | *Proteobacteria* | *Gammaproteobacteria* | NA | NA | NA | 0.33 | 0.06 | **5.49** | 0.03 | 0.07 | 0.35 |
| ASV_23625 | *Proteobacteria* | *Alphaproteobacteria* | *Rickettsiales* | *Anaplasmataceae* | NA | 0.32 | 0.06 | **5.37** | 0.00 | 0.00 | **11.31** |
| ASV_24536 | *Proteobacteria* | *Gammaproteobacteria* | NA | NA | NA | 0.33 | 0.06 | **5.49** | 0.03 | 0.07 | 0.35 |
| ASV_24537 | *Proteobacteria* | NA | NA | NA | NA | 0.33 | 0.06 | **5.49** | 0.03 | 0.07 | 0.35 |
| ASV_25792 | *Armatimonadetes* | *Chthonomonadetes* | *Chthonomonadales* | *Chthonomonadaceae* | *Chthonomonas/*  *Armatimonadetes_gp3* | 0.33 | 0.06 | **5.49** | 0.03 | 0.07 | 0.35 |
| ASV_25796 | *Planctomycetes* | *Planctomycetacia* | *Planctomycetales* | *Gemmataceae* | NA | 0.34 | 0.06 | **5.59** | 0.03 | 0.08 | 0.34 |
| ASV_25797 | *Proteobacteria* | *Gammaproteobacteria* | NA | NA | NA | 0.34 | 0.06 | **5.59** | 0.03 | 0.08 | 0.34 |
| ASV_27231 | *Proteobacteria* | *Deltaproteobacteria* | *Myxococcales* | NA | NA | 0.33 | 0.06 | **5.38** | 0.03 | 0.08 | 0.36 |
| ASV_27234 | *Acidobacteria* | *Acidobacteria_Gp17* | *Gp17* | NA | NA | 0.33 | 0.06 | **5.49** | 0.03 | 0.07 | 0.35 |
| ASV_27235 | *Proteobacteria* | *Gammaproteobacteria* | NA | NA | NA | 0.33 | 0.06 | **5.49** | 0.03 | 0.07 | 0.35 |
| ASV_27236 | *Proteobacteria* | *Alphaproteobacteria* | *Rhizobiales* | NA | NA | 0.33 | 0.06 | **5.49** | 0.03 | 0.07 | 0.35 |
| ASV_28825 | *Chloroflexi* | *Ktedonobacteria* | *Ktedonobacterales* | *Dictyobacteraceae* | *Dictyobacter* | 0.32 | 0.06 | **5.37** | 0.00 | 0.00 | **11.31** |
| ASV_28827 | *Planctomycetes* | *Planctomycetacia* | *Planctomycetales* | *Gemmataceae* | NA | 0.32 | 0.06 | **5.37** | 0.00 | 0.00 | **11.31** |
| ASV_29039 | *Actinobacteria* | NA | NA | NA | NA | 0.33 | 0.06 | **5.49** | 0.03 | 0.07 | 0.35 |
| ASV_29040 | *Armatimonadetes* | *Armatimonadetes_gp5* | NA | NA | NA | 0.33 | 0.06 | **5.49** | 0.03 | 0.07 | 0.35 |
| ASV_29044 | *Parcubacteria* | NA | NA | NA | NA | 0.34 | 0.06 | **5.59** | 0.03 | 0.08 | 0.34 |
| ASV_30937 | *Proteobacteria* | *Gammaproteobacteria* | *Chromatiales* | NA | NA | 0.32 | 0.06 | **5.37** | 0.00 | 0.00 | **11.31** |
| ASV_31165 | *Proteobacteria* | NA | NA | NA | NA | 0.33 | 0.06 | **5.38** | 0.03 | 0.08 | 0.36 |
| ASV_31167 | *Verrucomicrobia* | *Verrucomicrobiae* | *Verrucomicrobiales* | *Verrucomicrobiaceae* | *Phragmitibacter* | 0.33 | 0.06 | **5.49** | 0.03 | 0.07 | 0.35 |
| ASV_31169 | *Proteobacteria* | *Alphaproteobacteria* | *Caulobacterales* | *Caulobacteraceae* | NA | 0.33 | 0.06 | **5.49** | 0.03 | 0.07 | 0.35 |
| ASV_31177 | *Proteobacteria* | *Deltaproteobacteria* | *Myxococcales* | NA | NA | 0.34 | 0.06 | **5.59** | 0.03 | 0.08 | 0.34 |
| ASV_31178 | *Proteobacteria* | *Gammaproteobacteria* | NA | NA | NA | 0.34 | 0.06 | **5.59** | 0.03 | 0.08 | 0.34 |
| ASV_31179 | *Planctomycetes* | *Planctomycetacia* | *Pirellulales* | NA | NA | 0.34 | 0.06 | **5.59** | 0.03 | 0.08 | 0.34 |
| ASV_33739 | *Proteobacteria* | *Gammaproteobacteria* | NA | NA | NA | 0.32 | 0.06 | **5.37** | 0.00 | 0.00 | **11.31** |
| ASV_33740 | *Proteobacteria* | *Gammaproteobacteria* | NA | NA | NA | 0.32 | 0.06 | **5.37** | 0.00 | 0.00 | **11.31** |
| ASV_34133 | *Parcubacteria* | NA | NA | NA | NA | 0.33 | 0.06 | **5.38** | 0.03 | 0.08 | 0.36 |
| ASV_34134 | *Firmicutes* | *Clostridia* | *Clostridiales* | *Ruminococcaceae* | NA | 0.33 | 0.06 | **5.38** | 0.03 | 0.08 | 0.36 |
| ASV_34140 | *Acidobacteria* | *Acidobacteria_Gp1* | NA | NA | NA | 0.33 | 0.06 | **5.49** | 0.03 | 0.07 | 0.35 |
| ASV_34142 | *Chloroflexi* | *Anaerolineae* | *Anaerolineales* | *Anaerolineaceae* | NA | 0.33 | 0.06 | **5.49** | 0.03 | 0.07 | 0.35 |
| ASV_34143 | *Proteobacteria* | *Gammaproteobacteria* | NA | NA | NA | 0.33 | 0.06 | **5.49** | 0.03 | 0.07 | 0.35 |
| ASV_34144 | *Proteobacteria* | NA | NA | NA | NA | 0.33 | 0.06 | **5.49** | 0.03 | 0.07 | 0.35 |
| ASV_34158 | *Firmicutes* | *Clostridia* | *Clostridiales* | NA | NA | 0.34 | 0.06 | **5.59** | 0.03 | 0.08 | 0.34 |
| ASV_38323 | *Proteobacteria* | *Alphaproteobacteria* | NA | NA | NA | 0.32 | 0.06 | **5.37** | 0.00 | 0.00 | **11.31** |
| ASV_38325 | *candidate_division_*  *WPS-1* | NA | NA | NA | NA | 0.32 | 0.06 | **5.37** | 0.00 | 0.00 | **11.31** |

^*^ASV for which narrow-sense heritability (h^2^) > 0.2; SE: standard error; Z:z-score.

**Table S8** Fungal ASVs with significant broad-sense heritability (H^2^) > 0.2 across 132 different genotypes of Pinus radiata from 28 full-sib families (n = 505).

| **ASV** | **Phylum** | **Class** | **Order** | **Family** | **Genus** | **Species** | **H^2^** | **SE(H^2^)** | **Z(H^2^)** | **h^2^** | **SE(h^2^)** | **Z(h^2^)** |
| --- | --- | --- | --- | --- | --- | --- | --- | --- | --- | --- | --- | --- |
| ASV_499 **^a^** | *Ascomycota* | *Leotiomycetes* | *Helotiales* | *Myxotrichaceae* | *Oidiodendron* | *maius* | 0.41 | 0.07 | **5.52** | 0.04 | 0.28 | 0.16 |
| ASV_1751 | *Basidiomycota* | *Agaricomycetes* | *Cantharellales* | *Ceratobasidiaceae* | NA | NA | 0.23 | 0.05 | **4.70** | 0.00 | 0.00 | **16.19** |
| ASV_1914 | *Basidiomycota* | *Agaricomycetes* | *Thelephorales* | *Thelephoraceae* | *Tomentella* | *sublilacina* | 0.32 | 0.06 | **5.41** | 0.00 | 0.00 | **11.27** |
| ASV_2436 | *Basidiomycota* | *Agaricomycetes* | *Polyporales* | *Podoscyphaceae* | *Hypochnicium* | *NA* | 0.25 | 0.05 | **5.13** | 0.00 | 0.00 | **15.50** |
| ASV_2854 **^a^** | *Ascomycota* | *Eurotiomycetes* | *Eurotiales* | *Aspergillaceae* | *Aspergillus* | *inflatus* | 0.33 | 0.05 | **6.53** | 0.00 | 0.00 | **13.50** |
| ASV_3220 | *Ascomycota* | NA | NA | NA | NA | NA | 0.30 | 0.05 | **6.20** | 0.00 | 0.00 | **14.16** |
| ASV_3371 | *Basidiomycota* | *Agaricomycetes* | *Agaricales* | NA | NA | NA | 0.34 | 0.06 | **5.61** | 0.00 | 0.06 | 0.00 |
| ASV_3766 | *Ascomycota* | NA | NA | NA | NA | NA | 0.21 | 0.05 | **4.31** | 0.01 | 0.07 | 0.13 |
| ASV_4052 | *Basidiomycota* | *Agaricomycetes* | *Agaricales* | *Agaricaceae* | NA | NA | 0.34 | 0.06 | **5.61** | 0.00 | 0.06 | 0.00 |
| ASV_4121 | *Kickxellomycota* | *Kickxellomycetes* | *Kickxellales* | *Kickxellaceae* | *Spiromyces* | *aspiralis* | 0.34 | 0.06 | **5.61** | 0.00 | 0.06 | 0.00 |
| ASV_4374 | *Basidiomycota* | *Agaricomycetes* | *Hymenochaetales* | NA | NA | NA | 0.32 | 0.06 | **5.41** | 0.00 | 0.00 | **11.27** |
| ASV_5262 | *Ascomycota* | *Leotiomycetes* | *Helotiales* | *Helotiales* (family *Incertae sedis*) | *Cadophora* | *orchidicola* | 0.34 | 0.06 | **5.61** | 0.00 | 0.06 | 0.00 |
| ASV_5515 | *Basidiomycota* | NA | NA | NA | NA | NA | 0.32 | 0.06 | **5.41** | 0.00 | 0.00 | **11.27** |

**There were no ASVs for which narrow-sense heritability (h^2^) > 0.2; SE: standard error; Z: z-score**

### **
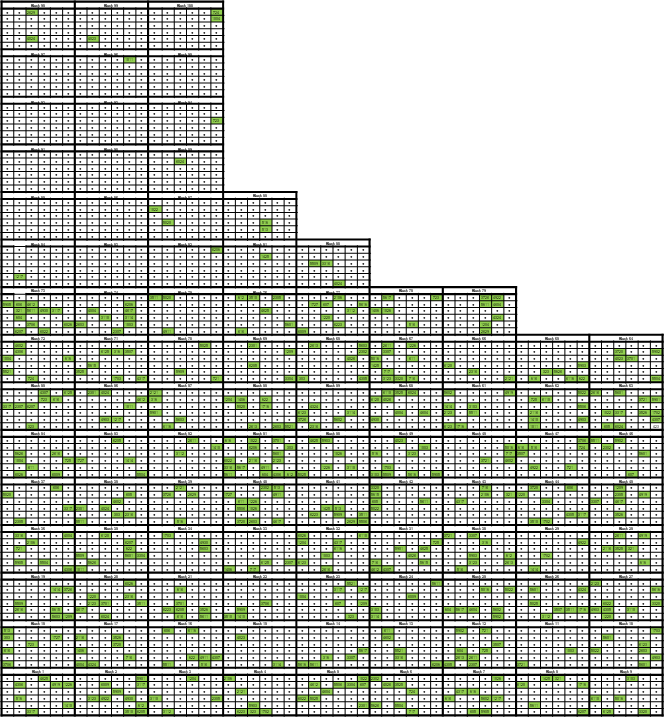
**Supplementary figures

**Figure S1** Layout of BC52_1 Pinus radiata genetic trial highlighting individuals sampled (n = 528); unselected trees are indicated by solid points. Spaces indicating block IDs are for illustrative purposes only, with 5 m (columns) and 2.5 m (rows) spacings between trees throughout the trial.

*
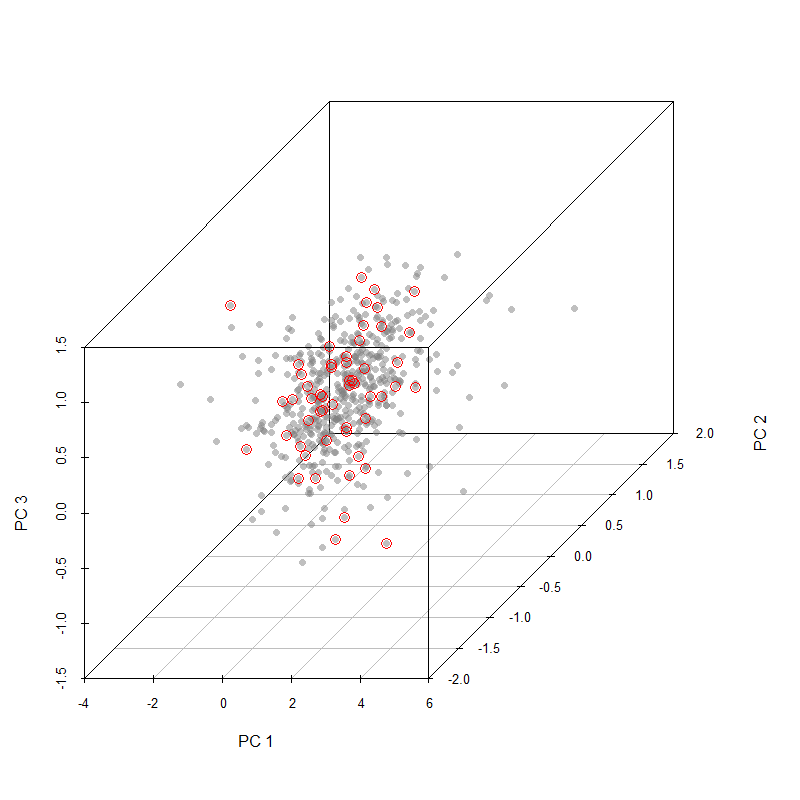
*

**Figure S2** Identification of soil samples (n = 53) for verification of diffuse reflectance mid-infrared spectroscopy (DRIFTS) predictions using conditioned Latin hypercube sampling (cLHS). Principal component analysis (PCA) was performed on the averaged spectra (n = 527, truncated to 600-4000 wavenumbers and baseline transformed).


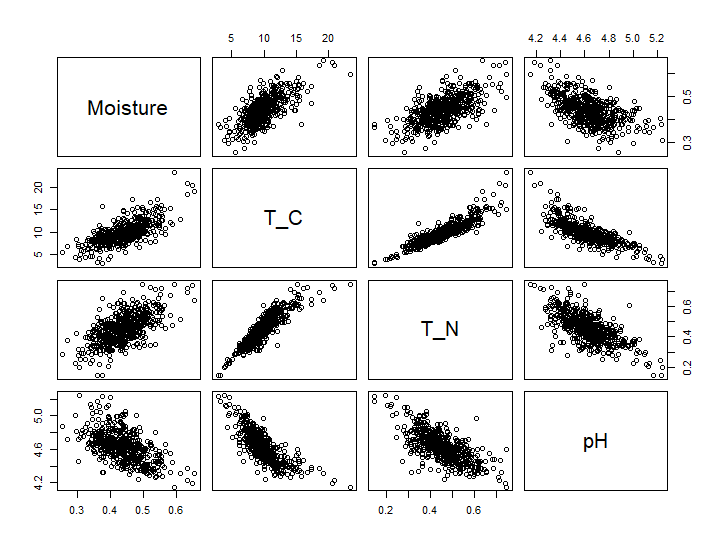


**Figure S3** Correlations among soil properties sampled across Pinus radiata genetic trial BC52_1 (n = 528), predicted using diffuse reflectance mid-infrared spectroscopy (DRIFTS). Properties include gravimetric moisture content (Moisture), total carbon (T_C), total nitrogen (T_N), and pH.


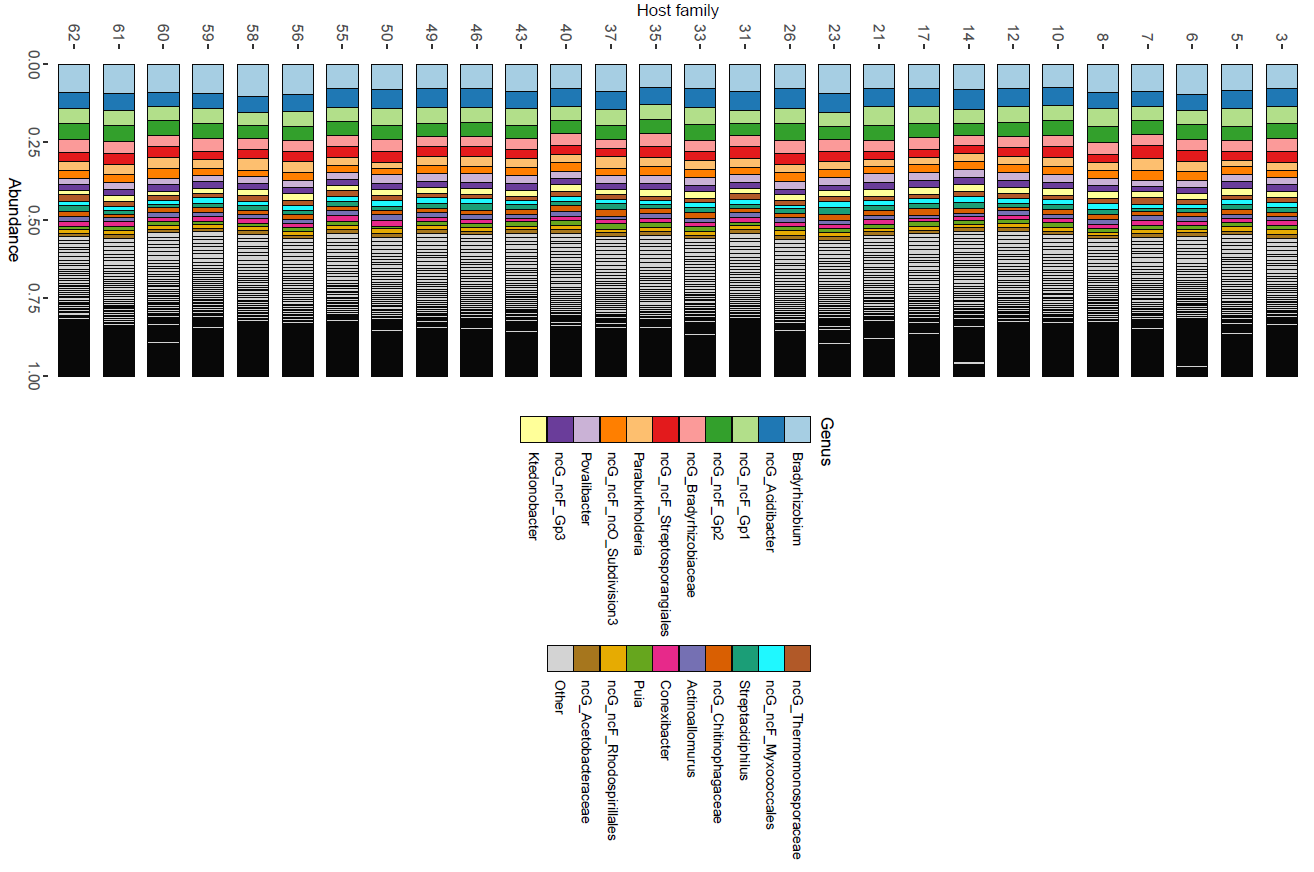

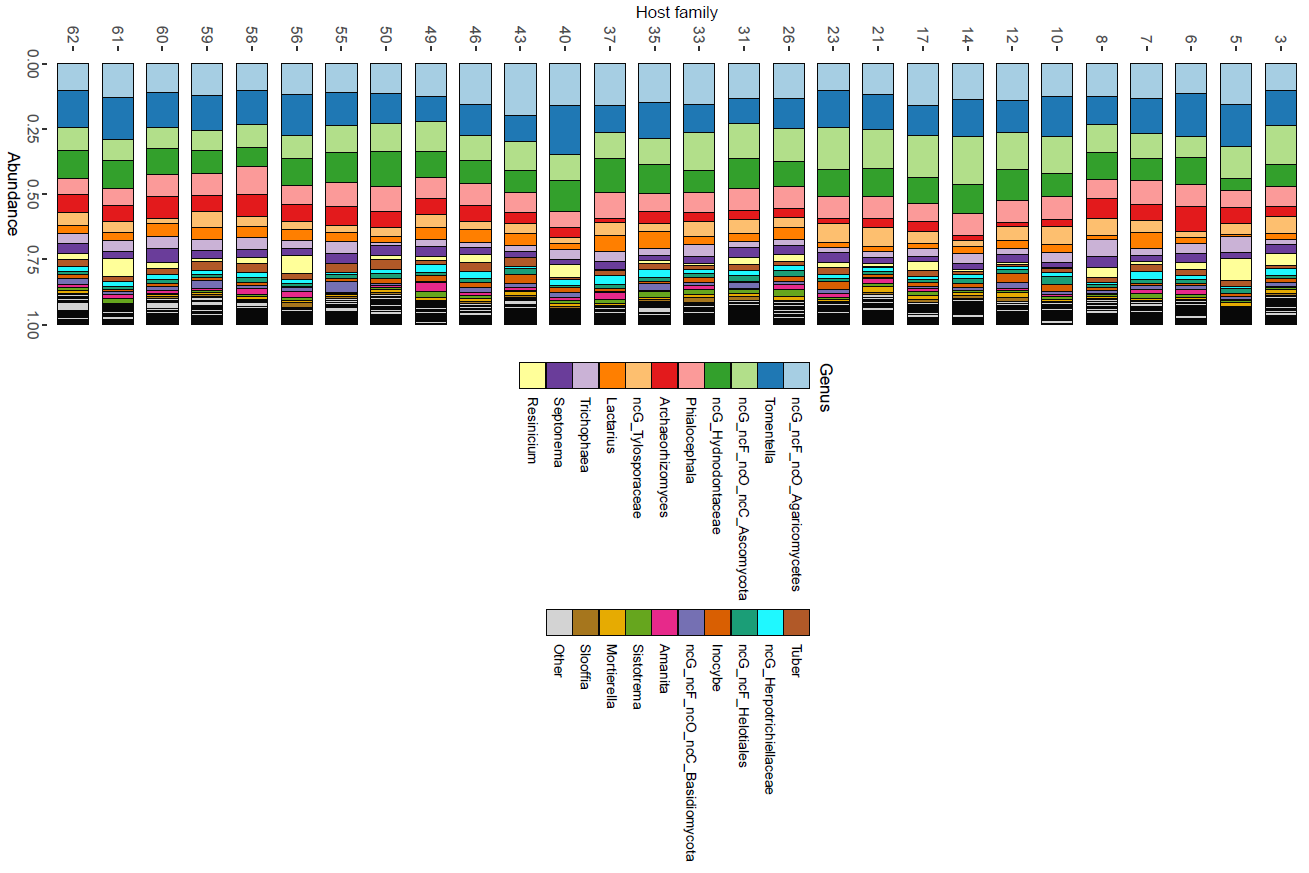


(a) Bacteria

(b) Fungi

**Figure S4** Relative abundance of the top twenty (a) bacterial and (b) fungal classes comprising the root microbiome of 28 full-sib families of Pinus radiata. Numbers on the Y axis indicate the host family, comprising three to six progeny each, with a total of 132 unique genotypes with four clonal replicates each.
